# Sex differences in the association between diabetes and risk of cardiovascular disease, cancer, and all-cause and cause-specific mortality: a systematic review and meta-analysis of 5,162,654 participants

**DOI:** 10.1186/s12916-019-1355-0

**Published:** 2019-07-12

**Authors:** Yafeng Wang, Adrienne O’Neil, Yurui Jiao, Lijun Wang, Jingxin Huang, Yutao Lan, Yikun Zhu, Chuanhua Yu

**Affiliations:** 1 Department of Epidemiology and Biostatistics, School of Health Sciences, Wuhan University, 185 Donghu Road, Wuchang District, Wuhan, 430071 China; 2 Melbourne School of Population and Global Health, University of Melbourne, Carlton, Australia; 3 Department of Endocrinology, Second Clinical Medical College, Shanxi Medical University, 382 Wuyi Road, Taiyuan, 030001 China; 4 Department of Neurology, Renji Hospital, School of Medicine, Shanghai Jiaotong University, Shanghai, China; 5 School of Nursing, Guangdong Pharmaceutical University, 283 Jianghai Avenue, Haizhu District, Guangzhou, China; 6 Global Health Institute, Wuhan University, Wuhan, China

**Keywords:** Diabetes, Sex difference, Mortality, Meta-analysis

## Abstract

**Background:**

Studies have suggested sex differences in the mortality rate associated with diabetes. We conducted a meta-analysis to estimate the relative effect of diabetes on the risk of all-cause, cancer, cardiovascular disease (CVD), infectious disease, and respiratory disease mortality in women compared with men.

**Methods:**

Studies published from their inception to April 1, 2018, identified through a systematic search of PubMed and EMBASE and review of references. We used the sex-specific RRs to derive the women-to-men ratio of RRs (RRR) and 95% CIs from each study. Subsequently, the RRR for each outcome was pooled with random-effects meta-analysis weighted by the inverse of the variances of the log RRRs.

**Results:**

Forty-nine studies with 86 prospective cohorts met the inclusion criteria and were eligible for analysis. The pooled women-to-men RRR showed a 13% greater risk of all-cause mortality associated with diabetes in women than in men (RRR 1.13, 95% CI 1.07 to 1.19; *P* < 0.001). The pooled multiple-adjusted RRR indicated a 30% significantly greater excess risk of CVD mortality in women with diabetes compared with men (RRR 1.30, 95% CI 1.13 to 1.49; *P* < 0.001). Compared with men with diabetes, women with diabetes had a 58% greater risk of coronary heart disease (CHD) mortality, but only an 8% greater risk of stroke mortality (RRR_CHD_ 1.58, 95% CI 1.32 to 1.90; *P* < 0.001; RRR_stroke_ 1.08, 95% CI 1.01 to 1.15; *P* < 0.001). However, no sex differences were observed in pooled results of populations with or without diabetes for all-cancer (RRR 1.02, 95% CI 0.98 to 1.06; *P* = 0.21), infectious (RRR 1.13, 95% CI 0.90 to 1.38; *P* = 0.33), and respiratory mortality (RRR 1.08, 95% CI 0.95 to 1.23; *P* = 0.26).

**Conclusions:**

Compared with men with the same condition, women with diabetes have a 58% and 13% greater risk of CHD and all-cause mortality, respectively, although there was a significant heterogeneity between studies. This points to an urgent need to develop sex- and gender-specific risk assessment strategies and therapeutic interventions that target diabetes management in the context of CHD prevention.

**Electronic supplementary material:**

The online version of this article (10.1186/s12916-019-1355-0) contains supplementary material, which is available to authorized users.

## Background

According to the Global Burden of Disease Study (GBD), non-communicable diseases (NCDs) are the main cause of premature deaths amongst the world’s population [1]. As one of four main NCDs, diabetes affected an estimated 387 million people throughout the world and caused around 1.3 million deaths worldwide in 2010 alone [2–4]. With the increasing prevalence of physical inactivity and obesity, the burden of diabetes is predicted to increase to 592 million by 2035, making it a major contributor to the global burden of disease [5].

Type 2 diabetes mellitus is associated with an approximate twofold increase in the risk of all-cause mortality as well as death from cardiovascular disease (CVD), kidney disease, infectious disease, respiratory disease, and several specific forms of cancer [6]. Previous meta-analyses, through internal, within-study comparisons of female and male participants, have observed that women with diabetes are at substantially higher risk of coronary heart disease (CHD), stroke, and gastric cancer compared to affected men. On the other hand, no sex differences were found between diabetes and the risk of esophageal cancer, colorectal cancer, and pancreatic cancer [7–9]. However, the magnitude of the excess risk of these and other cause-specific outcomes that are conferred by diabetes for men and women is unknown. Furthermore, it is unclear whether important confounders (e.g., age) and methodological heterogeneity (duration of follow-up, method of diabetes classification or assessment) would modify any such sex differential in the association between diabetes and mortality. It is also unclear whether such a difference might be more pronounced in recent years with the growing obesity epidemic (e.g., year of publication).

Accordingly, we sought to conduct a meta-analysis of prospective cohort studies in order to (i) calculate any sex differential in the association between diabetes and risk of cardiovascular disease, cancer, and all-cause and cause-specific mortality for the general population and (ii) to determine whether these associations are modified by demographics, setting, length of follow-up, diabetes measurement, and recency of publication.

## Methods

### Search strategy

The meta-analysis was performed in accordance with the Meta-Analysis of Observational Studies in Epidemiology guidelines [10] and the Preferred Reporting Items for Systematic Reviews and Meta-Analyses statement [11] (Additional file 1: Table S1). We searched the PubMed and EMBASE databases from their inception to April 1, 2018. Details of the search strategy using a combined text word and medical subject heading are displayed in Additional file 1. The articles were restricted to English language studies. Moreover, the reference lists of the retrieved publications and reviews were checked for other potentially relevant studies.

### Study selection

Studies were included if they met the following criteria: (1) the study was a prospective cohort design; (2) the outcomes included all-cause mortality, cancer mortality, CVD mortality, CHD mortality, stroke mortality, infectious disease mortality, and/or respiratory disease mortality; (3) the studies provided odds ratio (OR), relative risk (RR), or hazard ratio (HR) with 95% confidence intervals (CI) for the associations between diabetes and mortality disaggregated for men and women participants; and (4) when multiple publications reported on the same population or subpopulation, the study with the most recent or most informative data was included. The exclusion criteria were as follows: (1) matched prospective cohort study design, (2) studies reporting only estimates for type 1 diabetes, (3) studies not adjusting for age, and (4) studies of populations that predominantly consisted of individuals with underlying pathological disorders, such as cardiovascular disease or cancer. We also used individual participant data from the America’s National Health Interview Surveys (1997 to 2009) linked to the National Death Index records through December 31, 2011. Extensive details about the questionnaire, methodology, data, and documentation are available on the NHIS website. [https://www.cdc.gov/nchs/nhis/about_nhis.htm].

### Data extraction and study quality assessment

Two investigators (YFW and YRJ) independently reviewed all potentially eligible studies using predefined criteria and extracted the data from each paper. In case of incomplete or unclear data, the authors were contacted where possible. The cohort study quality was estimated using the nine-star Newcastle-Ottawa quality assessment Scale (NOS) ranging from zero to nine stars [12]. Disagreements were resolved by consensus between the authors.

### Statistical analysis

The RR was used as a measure of the association between diabetes and outcome risk. For individual participant data, we used Cox proportional hazards regression to obtain HRs (regarded as RRs). If the included studies did not report the RRs, the HRs were directly considered as RRs and the ORs were converted into RRs using the formula: RR = OR/[(1 − Po) + (OR × Po)], in which Po was the incidence of the outcome of interest in the non-diabetes group [13]. For studies that reported RRs in different age groups, we pooled these RRs with inverse variance random-effect models, and then we used combined estimates for that study. For the primary analysis, we used the sex-specific RRs to derive the women-to-men ratio of RRs (RRR) and 95% CIs from each study, as previously described [14]. Subsequently, the RRR for each outcome was pooled with random-effects meta-analysis weighted by the inverse of the variances of the log RRRs. We also pooled RRs for men and women separately, using an identical approach. The heterogeneity among the included studies was evaluated by the *Q* test and *I*^2^ statistic [15].

Subsequently, where the number of included studies was more than 10 for each outcome of interest, sensitivity analyses were performed by mean age (≤ 60 versus > 60 years), region (Asia versus Europe versus America versus others), publish year (≤ 2000 versus 2001–2009 versus ≥ 2010), length of follow-up (≤ 10 versus > 10 years), and ascertainment of diabetes (known diabetes versus newly diagnosed diabetes versus both). Random-effects meta-regression analyses were used to evaluate whether the differences in the mean/medium duration of study follow-up and mean age of participants at baseline contributed to the heterogeneity between the studies. Publication bias was assessed by Begg’s rank correlation test and its funnel plots of the natural log of the RRR against its standard error [16]. Where publication bias was detected, trim and fill analyses were used to adjust the RRs or ratio of RRs. All statistical analyses were performed with Stata version 13.0 (StataCorp, College Station, TX, USA).

## Results

**Fig. 1:**
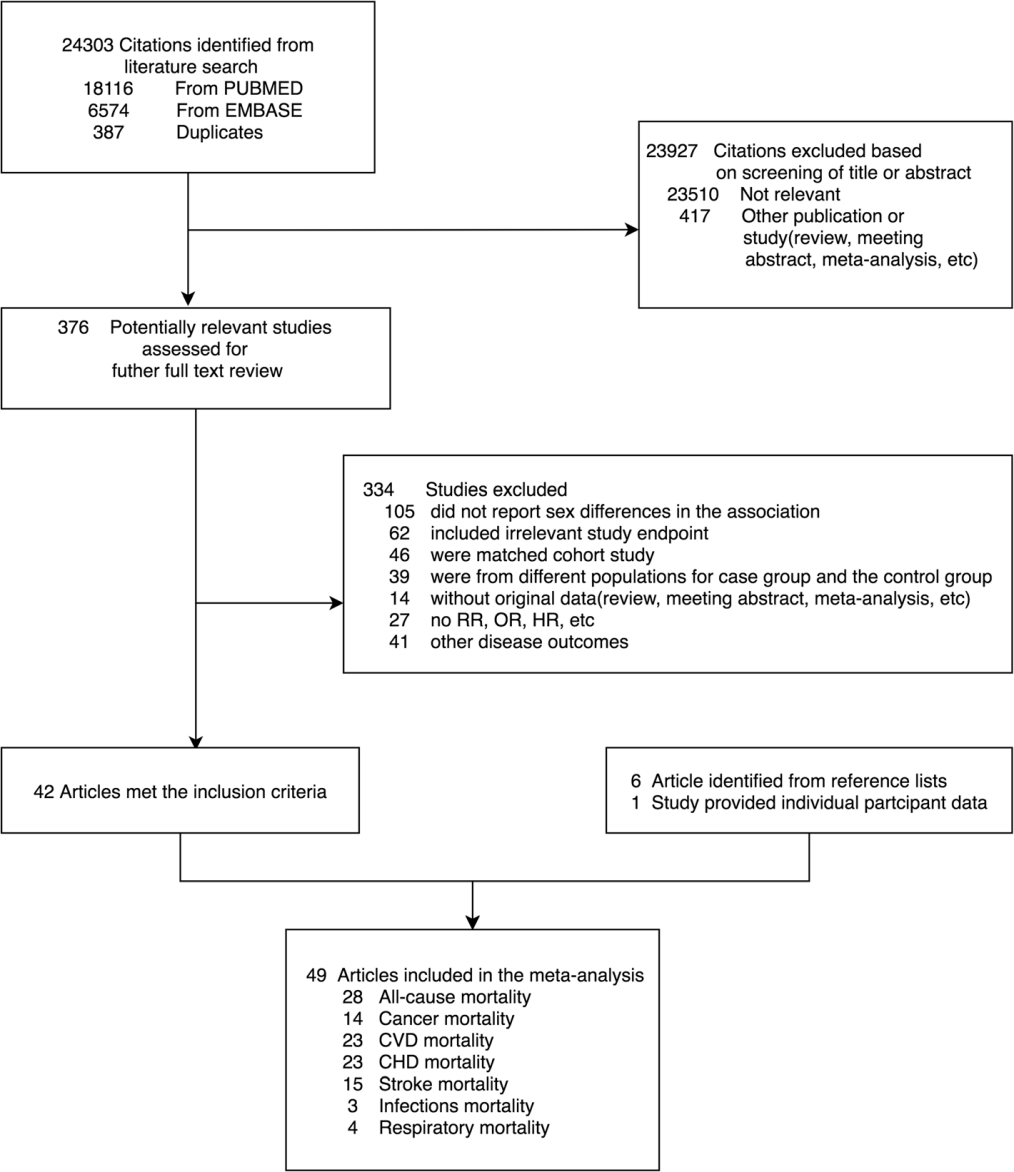
Flowchart for study selection for the meta-analysis

Of the 24,303 references identified through the systematic search, 375 were examined in the full-text review (Fig. 1). In addition, 6 articles were retrieved from the reference lists of relevant articles and reviews. Subsequently, individual participant data from NHIS were added to these published results. Finally, 49 studies with 86 prospective cohorts met the inclusion criteria and [17–63] were eligible for analysis (Table 1).

**Table 1:** Characteristics of studies included in the meta-analysis

Author	Study location	Study name	Mean baseline age (years)	No. of participants	No. of diabetes	Ascertainment of diabetes	Outcome	Mean follow-up years	Maximum adjustment available
Jousilahti et al. 1999 [17]	Finland	Pekka et al-Finnish	44.4	14,786	NA	Self-reported	CHD mortality	NA	Age, study year, area, smoking, HDL, HDL/cholesterol ratio, SBP, BMI
Oba et al. 2008 [18]	Japan	Takayama study	54.6	29,079	1217	Self-reported	All-cause mortality, cancer mortality, CVD mortality, CHD mortality, stroke mortality	7	Age, smoking, BMI, physical activity, length of education in years, history of hypertension, total energy intake, intake of vegetables, fat, and alcohol
Hu et al. 2005 [19]	Finland	Kuopio and North Karelia study	44.3	50,281	962	Self-reported	All-cause mortality, CVD mortality, CHD mortality, stroke mortality	17.2	Age, study year, BMI, SBP, TC, smoking
Madssen et al. 2012 [20]	Norway	HUNT 1 study	74.6	47,586	2421	Self-reported, measured	CHD mortality	NA	Age, BMI, hypertension, CVD, smoking, physical activity
DECODE Study Group 2001 [21]	Europe	DECODE Study	53.2	22,514	1807	Self-reported, measured	All-cause mortality, CVD mortality, CHD mortality, stroke mortality	8.8	Age, center, TC, BMI, SBP, smoking
Keli et al. 1993 [22]	USA	Charleston Heart Study	50	2181	NA	Self-reported	All-cause mortality, CHD mortality	30	Age, SBP, serum cholesterol, smoking, BMI, years of education, history of diabetes
Friberg et al. 2004 [23]	Denmark	Copenhagen City Heart Study	58.4	29,310	1072	Self-reported, measured	CVD mortality	4.7	Age, AF, arterial hypertension, SBP, MI, ELVH, smoking, FEV2
VR et al. 1996 [24]	Pacific island nation of Fiji	NA	50.5	2546	2638	Self-reported, measured	All-cause mortality, CVD mortality, CHD mortality, stroke mortality	11	Age, SBP, BMI, TC, smoking, survey area
Bozorgmanesh et al. 2011 [25]	Iran	Tehran Lipid and Glucose Study	47	6331	897	Self-reported, measured	All-cause mortality	8.6	Age, smoking, SBP, WC, TC, TG, HDL-C, non-HDL-C, CVD, intervention
Kleinman et al. 1988 [26]	USA	First National Health and Nutrition Examination Survey	58.1	7381	407	Self-reported, measured	All-cause mortality, CVD mortality, CHD mortality	10	Age, SBP, serum cholesterol, BMI, smoking
Magliano et al. 2010 [27]	Mauritius	NA	40.9	9559	NA	Self-reported, measured	All-cause mortality, CVD mortality	15	Age, WC, HIP, smoking, hypertension, ethnicity, CVD, education, HDL-C, TG, TC
Elizabeth et al. 1991 [28]	USA	The Rancho Bernardo Study	61.9	2471	334	Self-reported, measured	CHD mortality	14.4	Age, SBP, cholesterol, BMI, smoking
Fraser et al. 1992 [29]	USA	The Adventist Health Study	52.8	27,658	NA	Self-reported	CHD mortality	6	Age, hypertension, smoking, physical activity, BMI
Sievers et al. 1992 [30]	India	NA	49.5	5131	1266	Measured	All-cause mortality, cancer mortality, IHD mortality, stroke mortality, infections mortality	10	Age
Seeman et al. 1993 [31]	USA	The New Haven EPESE cohort	NA	2812	386	Self-reported	CHD mortality	6	Age, education, BMI, smoking, alcohol, vegetable intake, red meat intake, physical activity, aspirin use
Campbell et al. 2012 [6]	USA	Cancer Prevention Study-II	NA	1,053,831	52,655	Self-reported	All-cause mortality, cancer mortality, CVD mortality, CHD mortality, stroke mortality, respiratory system mortality, infections mortality	26	Age, high blood pressure, BMI, smoking, elevated serum cholesterol, elevated serum triglycerides, elevated serum uric acid, IGT, obesity, hyperuricemia
Wang et al. 2012 [32]	Taiwan	Taiwan Survey of Hypertension, Hyperglycemia, and Hyperlipidemia	45.6	4289	335	Measured	All-cause mortality, CVD mortality	7.7	Age, education, marital status, housing tenure, car ownership
Natarajan et al. 2003 [33]	USA	Framingham Heart Study and the Framingham Offspring Study	52.2	5243	229	Measured	CHD mortality	20	Age, chest pain on exertion, BP, use of anti-hypertensive medication, smoking, BMI
Vilbergsson et al. 1998 [34]	Iceland	The Reykjavik Study	52.8	18,912	477	Self-reported, measured	All-cause mortality, CVD mortality	17	Age strata, CAD, stroke, BMI, alcohol, smoking, betel nut chewing, physical activity, income
Qvist et al. 1996 [35]	Sweden	NA	59.1	5306	NA	Self-reported	CVD mortality, stroke mortality	10	Age, smoking, hypertension, TC, HDL-C, BMI
Tunstall-Pedoe et al. 1997 [36]	England	Edinburgh and north Glasgow MONICA population surveys	49.5	11,629	NA	Self-reported	All-cause mortality, CHD mortality	7.6	Age, smoking, BMI, hypertension, TC, TG, calendar year
Nilsson et al. 1998 [37]	Sweden	Swedish Annual Level-of-Living Survey	NA	39,055	776	Self-reported	All-cause mortality, CVD mortality, CHD mortality, stroke mortality	16	Age
Imazu et al. 2002 [38]	USA	The Hawaii-Los Angeles-Hiroshima study	60.9	927	169	Measured	CVD mortality, CHD mortality	14	Age, BMI, serum uric acid, TC, TG, hypertension, ECG (abnormal Q), ECG (ST-T changes), smoking
Hart et al. 1999 [39]	England	The Renfrew/Paisley general population study	NA	15,406	NA	Self-reported	Stroke mortality	20	Age, DBP, smoking, FEV1, height, BMI, diabetes, preexisting CHD
Bragg et al. 2014 [40]	China	The China Kadoorie Biobank	51.5	512,869	512,869	Self-reported Measured	All-cause mortality, cancer mortality, IHD mortality, stroke mortality, respiratory disease mortality, infections mortality	7	Age, geographic area, education, smoking, alcohol, physical activity, BMI.
Kato et al. 2015 [41]	Japan	Japan Public Health Center-based prospective study	50.2	99,584	4286	Self-reported	All-cause mortality, cancer mortality, IHD mortality, stroke mortality	20	Age, BMI, alcohol, smoking, hypertension, physical activity, area
Johansen et al. 1987 [42]	Canada	The Nutrition Canada survey	NA	8094	NA	Self-reported	All-cause mortality	10	Age, respondent status, smoking, DBP, history of diabetes or presence of glucose in the urine, BMI, serum cholesterol level, alcohol consumption
Suemoto et al. 2014 [43]	Brazil	The SABE Study	71	1882	312	Self-reported	All-cause mortality	7	Age, race, marital status, years of education, childhood socioeconomic status, occupation, income, heart disease, lung disease, stroke, arthritis, depressive symptoms, alcohol, smoking, BMI, physical activity, frailty, nutritional status, year of entry in the study
Jee et al. 2005 [44]	Korea	The National Health Insurance Corp	46.9	1,298,358	62,924	Self-reported, measured	All-cause mortality, all-cancer mortality	10	Age, age squared, smoking, alcohol
Fraser et al. 1997 [45]	Spain	Non-Hispanic white Seventh-Day Adventists from California	NA	603	NA	Self-reported	All-cause mortality, CHD mortality	12	Age, smoking, physical activity, nuts per week, fruit per day, bread, sweet desserts per week, beef per week, fish per week
Moe et al. 2013 [46]	Norway	HUNT 2	46.5	53,587	1195	Self-reported, measured	CVD mortality	12	Age, physical activity, smoking, alcohol, education, BMI, SBP, TC
Liu et al. 2011 [47]	USA	The LSOA II study	80	9246	NA	Self-reported	All-cause mortality	8	Age, marital status, living arrangement, educational attainments, hypertension, CHD, stroke
Vimalananda et al. 2014 [48]	USA	The Cardiovascular Health Study	72.6	4817	681	Self-reported, measured	All-cause mortality	12.5	Age, clinical site, HDL-C, LDL-C, SBP, anti-hypertensive medication use, CRP
Eichner et al. 2010 [49]	USA	The Strong Heart Study	56	4293	265	Self-reported, measured	CVD mortality	17	Age, BMI, LDL-C, HDL-C, physical activity, hypertension, diabetes, macro- and microalbuminuria
Bozorgmanesh et al. 2012 [50]	Iran	The Tehran lipid and glucose study	33.3	8795	1449	Self-reported, measured	All-cause mortality, CVD mortality	9	Age, smoking, SBP, using antihypertensive drugs, TC, HDL-C
Moe et al. 2013 [51]	Norway	HUNT 1 study	47.9	56,170	1105	Self-reported	CVD mortality, IHD mortality	24	Age, birth, smoking, education, alcohol, SBP, BMI, physical activity
Kakehi et al. 2014 [52]	Japan	The Jichi Medical School Cohort Study	55.1	11,998	2706	Measured	All-cause mortality, cancer mortality, CVD mortality, stroke mortality	10.7	Age, BMI, SBP, TC, HDL-C, TC, smoking, alcohol
Shen et al. 2014 [53]	China	Elderly health centers in Hong Kong	69.5	66,820	9225	Self-reported	All-cause mortality, cancer mortality, CVD mortality, IHD mortality, stroke mortality, respiratory disease mortality, infectious disease mortality	12.5	Age, alcohol, smoking, physical activity, education, housing, monthly expenditure
Hiltunen et al. 2005 [54]	Finland	Kempele, Oulunsalo and Hailuoto study	76	379	98	Self-reported, measured	All-cause mortality	9.8	Age, BMI, CVD, hypertension, physical activity, self-rated health
Gordon-Dseagu et al. 2014 [55]	England	The Health Survey for England or Scottish Health Survey	47	204,533	7199	Self-reported	All-cause mortality, cancer mortality, CVD mortality	10	Age, sex, smoking, BMI
Yeh et al. 2012 [56]	USA	The CLUE II (Give Us a Clue to Cancer and Heart Disease) cohort	51.8	18,280	599	Treated diabetes	All-cancer mortality	17	Age, BMI, smoking, education level, hypertension treatment, and high cholesterol treatment, menopausal status, history of use of oral contraceptives, history of use of hormone replacement therapy
Chen et al. 2017 [57]	Asia	The ACC	53.9	771,297	NA	Self-reported	All-cancer mortality	12.7	Age, BMI, smoking, alcohol, educational attainment, urban residence
Zhou et al. 2010 [58]	Europe	The DECODE study	53.4	44,655	NA	Self-reported, measured	All-cancer mortality	21.4	Age, study cohort, BMI, SBP, cholesterol, smoking
Drake et al. 2017 [59]	Sweden	The MDCS	57.9	26,953	21,940	Self-reported	Cancer mortality	17	Age, calendar year of study entry, height, smoking, physical activity, alcohol, educational level, past food habit change, hypertension, use of lipid-age, lowering drugs, family history of cancer, BMI
Preis et al. 2009 [60]	USA	The Framingham Heart Study	58.1	10,333	NA	Self-reported, measured	All-cause mortality, CVD mortality	25	Age
NHIS	USA	NHIS	46.8	339,113	26,039	Self-reported	All-cause mortality, cancer mortality, CVD mortality, stroke mortality	6.6	Age, race, BMI, smoking, drinking, education level, hypertension, physical activity, marital status, CVD, cancer
Natarajan et al. 2005 [61]	USA	National Health and Nutrition Examination Survey Epidemiologic Follow-up Study	52.4	10,871	539	Measured	CHD mortality	NA	Age, race, smoking, hypertension, serum cholesterol level, body mass index
Hirakawa et al. 2017 [62]	Japan	EPOCH-JAPAN study	58.2	38,854	1867	Measured	All-cause mortality, CVD mortality, CHD mortality, stroke mortality	10.3	Age, SBP, serum total cholesterol, BMI, current smoking status, habitual alcohol intake
Alegre-Díaz et al. 2016 [63]	Mexico	Mexico City Study	51.7	146,046	17,411	Self-reported	All-cause mortality	12	Age, smoking, district, education level, height, weight, WC, Hip

*Abbreviations*: *BMI* body mass index (Quetelet index), *BP* blood pressure, *SBP* systolic blood pressure, *DBP* diastolic blood pressure, *TC* total cholesterol, *HDL* high-density lipoprotein, *HDL-C* high-density lipoprotein cholesterol, *LDL-C* low-density lipoprotein cholesterol, *TG* triglyceride, *TG/HDL-C* triglyceride-to-high-density lipoprotein cholesterol ratio, *ELVF* electrocardiographic left ventricular hypertrophy, *AF* atrial fibrillation, *MI* myocardial infarction, *FEV1* forced expiratory volume in 1 s, *FEV*_*2*_ forced expiratory volume in 2 s, *CVD* cardiovascular disease (angina, coronary heart disease, stroke, or amputation) (family history of premature CVD), *IHD* ischemic heart disease, *CHD* coronary heart disease, *WC* waist circumference, *Hip* hip circumference, *IGT* impaired glucose tolerance, *CRP* C-reactive protein, *NA* not available

The characteristics of the included studies are described in Table 1. Baseline surveys were conducted between 1950 and 2014, and the number of participants ranged from 379 to 1,298,358. The mean/median duration of follow-up ranged from 6.0 to 21.4 years, while the average baseline age was between 33.3 and 80.0 years. The quality of all included studies based on NOS was high (Additional file 1: Table S2). All studies adjusted for age and most of the studies also controlled for smoking (*n* = 77), hypertension (*n* = 71), and body mass index (*n* = 68).

Twenty-eight studies with 3,887,585 participants were included to assess the sex-specific association between diabetes and all-cause mortality. For cause-specific mortality, 14 studies with 4,482,501 reported on cancer mortality, 23 studies with 2,067,486 reported on CVD mortality, 23 studies with 2,050,929 reported on CHD mortality, 15 studies with 2,292,387 reported on stroke mortality, 4 studies with 1,633,520 reported on respiratory disease mortality, and 3 studies with 1,638,651 reported on infectious disease mortality.

### Sex-specific association between diabetes and risk of all-cause, cancer, CVD, infectious disease, and respiratory disease mortality

**Fig. 2:**
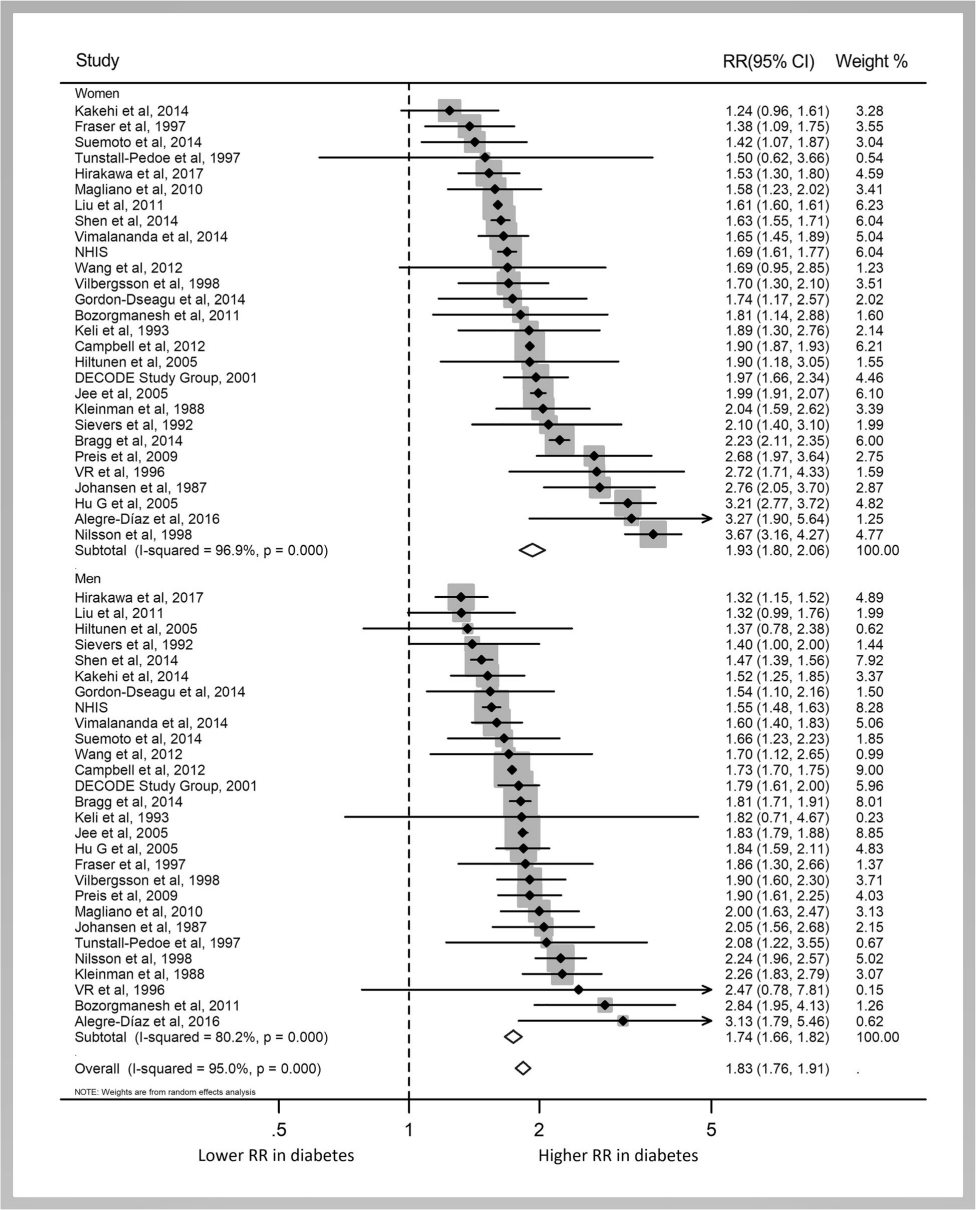
Pooled RRs for risk of all-cause mortality

**Fig. 3:**
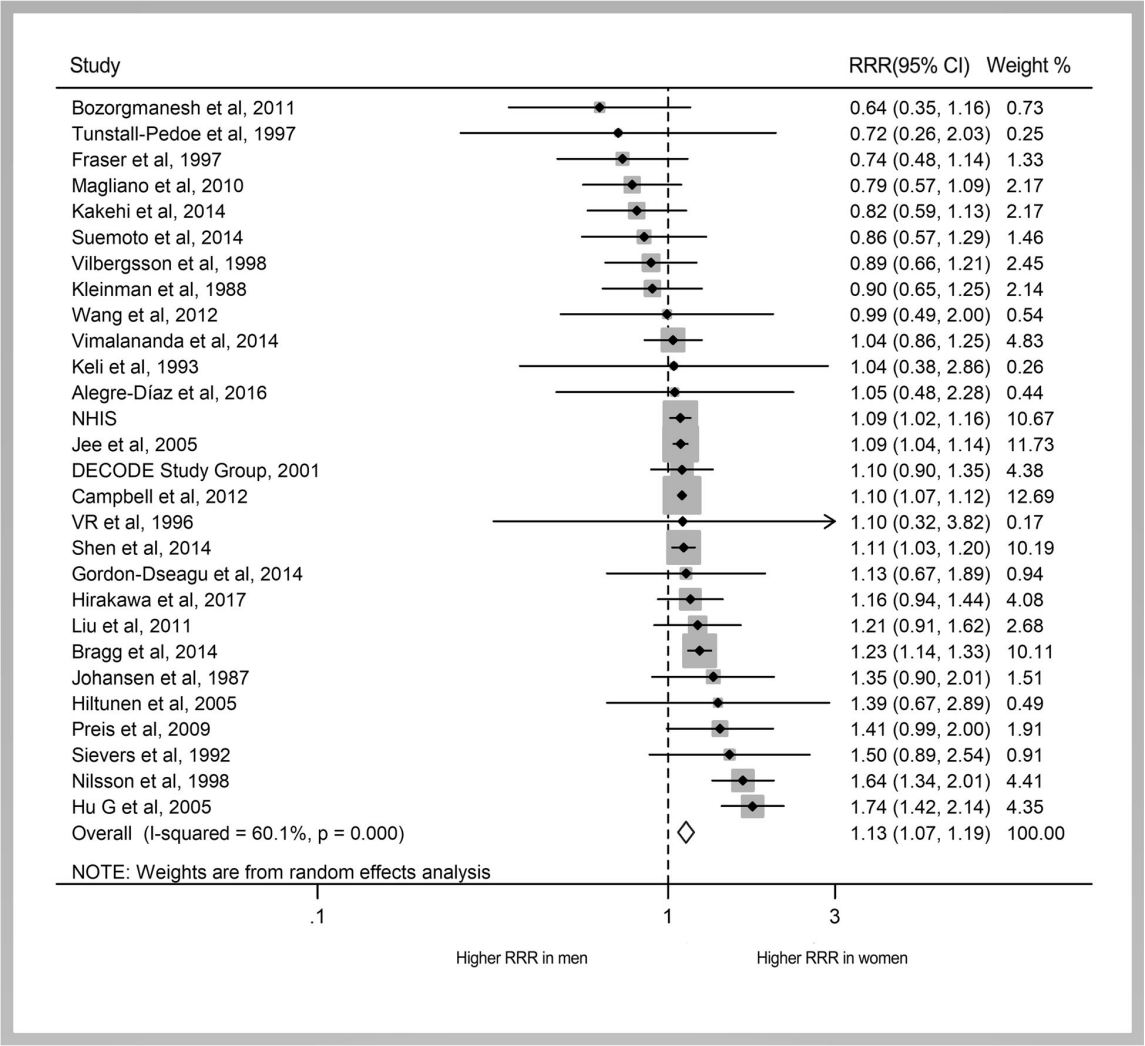
Pooled women-to-men RRRs for risk of all-cause mortality

**Fig. 4:**
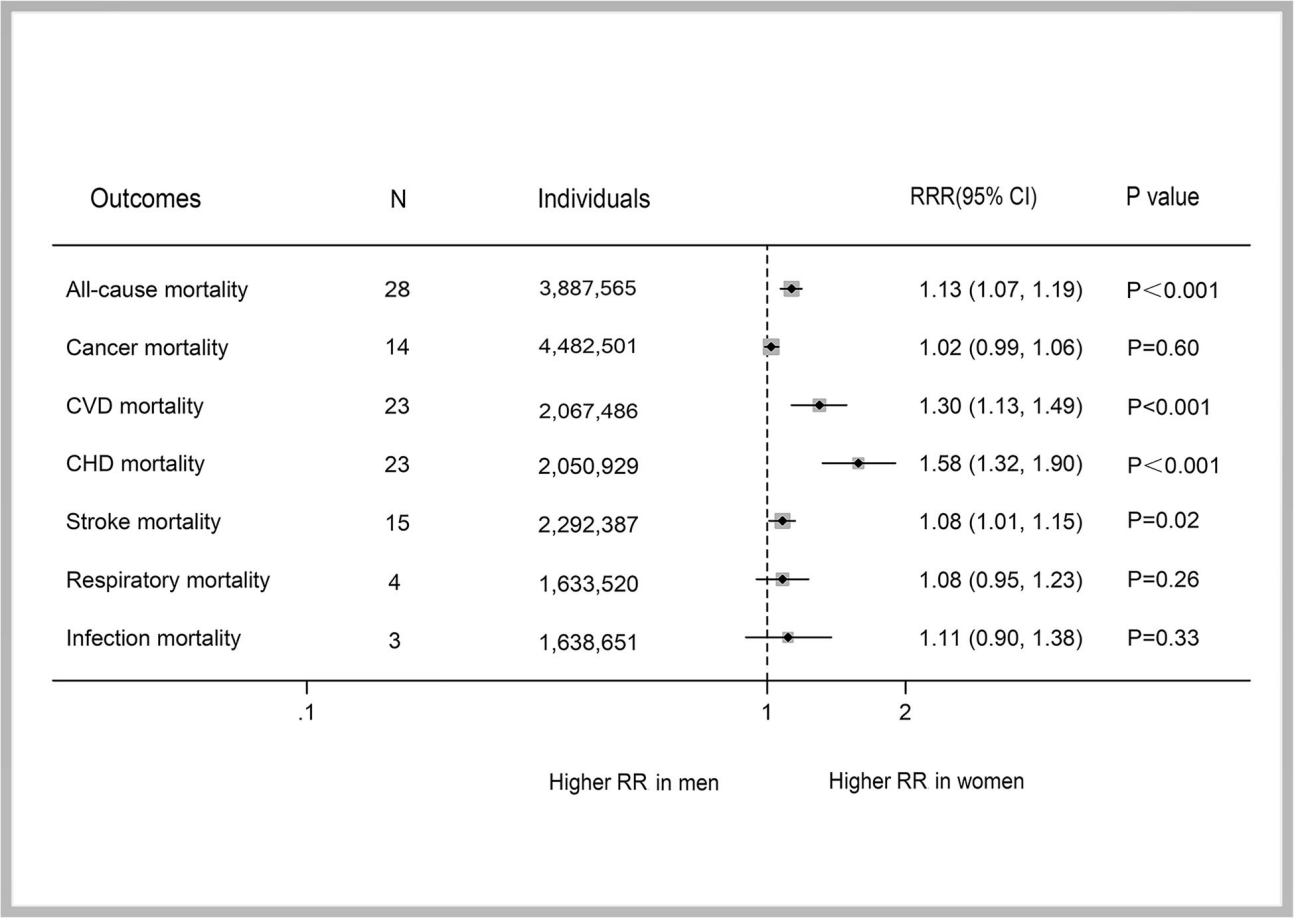
Pooled women-to-men RRRs for risk of all-cause, cancer, CVD, CHD, stroke, respiratory, and infectious mortality

The pooled multiple-adjusted RRs of all-cause mortality associated with diabetes compared with no diabetes were 1.93 (95% CI 1.80 to 2.06; Fig. 2) in women and 1.74 (1.67 to 1.82) in men. The pooled women-to-men RRR showed a 13% greater risk of all-cause mortality associated with diabetes in women than in men (RRR 1.13, 95% CI 1.07 to 1.19; *P* < 0.001; Figs. 3 and 4). There was, however, a significant heterogeneity between the studies (*I*^2^ = 60%, *P* < 0.001; Fig. 2).

The pooled multiple-adjusted RRs showed that diabetes was associated with a 26% (1.16 to 1.36) increased risk for cancer mortality in women and a 29% (1.18 to 1.42; Additional file 1: Figure S1) increased risk in men. There was no evidence of a sex difference in the association between diabetes and cancer mortality; the pooled multiple-adjusted RRR of cancer mortality for diabetes was 1.02 (0.98 to 1.06; *P* = 0.21; Fig. 4; Additional file 1: Figure S2). No evidence of significant between-study heterogeneity was found (*I*^2^ = 0%; *P* = 0.60).

Compared with unaffected individuals, the pooled RR for CVD mortality in people with diabetes was 2.42 (2.10 to 2.78; Additional file 1: Figure S3) in women and 1.86 (1.70 to 2.03) in men. Overall, the pooled multiple-adjusted RRR indicated a 30% significantly greater excess risk of CVD mortality in women with diabetes compared with men (RRR 1.30, 95% CI 1.13 to 1.49; *P* < 0.001; Fig. 4; Additional file 1: Figure S4), but with significant heterogeneity between the studies (*I*^2^ = 78%, *P* < 0.001). In addition, the pooled RR of CHD mortality for individuals with diabetes compared with those without diabetes was higher in women than in men [women, 3.16 (2.61 to 3.82); men, 2.11 (1.98 to 2.25); both *P* < 0.001; Additional file 1: Figure S5]. Compared with men with diabetes, women with diabetes had a 58% greater risk of CHD mortality, but only an 8% greater risk of stroke mortality [CHD mortality (RRR 1.58, 95% CI 1.32 to 1.90; *P* < 0.001; Additional file 1: Figure S6); stroke mortality (RRR 1.08, 95% CI 1.01 to 1.15; *P* < 0.001; Additional file 1: Figure S7); Fig. 4]. Moreover, there was no heterogeneity between the studies examining stroke mortality, but significant heterogeneity between the studies for CHD mortality [CHD mortality (*I*^2^ = 67%, *P* < 0.001); stroke mortality (*I*^2^ = 0%, *P* = 0.74)].

Compared with those without, women and men with diabetes had approximately 31% and 22% greater risk of respiratory disease mortality, respectively (Additional file 1: Figure S8). However, no sex differences were observed (RRR 1.08, 95% CI 0.95 to 1.23; *P* = 0.26; Fig. 4) nor significant heterogeneity (*I*^2^ = 0; *P* = 0.98).

Diabetes was associated with an approximately twofold increase in the risk of infectious disease-related mortality [women, 2.13 (1.89 to 2.42); men, 1.94 (1.66 to 2.26); both *P* < 0.001; (Additional file 1: Figure S9)]. There was no evidence of sex differences (RRR 1.11, 95% CI 0.90 to 1.38; *P* = 0.33; Fig. 4).

### Subgroup, meta-regression, and publication bias analyses

We performed subgroup analyses for cancer, CHD, stroke, CVD and all-cause mortality outcomes. Results showed no evidence of heterogeneity between the subgroups stratified by study characteristics including age, geographical location, duration of follow-up, publish year, and method of diabetes ascertainment (Table 2). For the method of diabetes ascertainment, sex differences for CVD, CHD, and all-cause mortality conferred by diabetes were only significant in self-reported diagnosis (all-cause mortality: RRR 1.17, 95% CI 1.07 to 1.27, *P* < 0.001; CVD mortality: RRR 1.20, 95% CI 1.02 to 1.42, *P* < 0.001; CHD mortality: RRR 1.52, 95% CI 1.20 to 1.92, *P* < 0.001). The pooled RRR for CHD, stroke, CVD, and all-cause mortality did not vary by mean age of the participants at baseline, mean/medium duration of follow-up, baseline prevalence of diabetes, and women-to-men ratio of diabetes prevalence (all *P* > 0.1). We found no evidence of publication bias for cancer, CHD, stroke, CVD, respiratory disease, infectious disease, and all-cause mortality (*P* > 0.10).

**Table 2:** Sensitivity analyses of women-to-men ratio of relative risks for the outcomes associated with diabetes

	Individuals	*N*	RRR	Lower	Upper	*P* value	Test for heterogeneity			*P* value for interaction
							*I*^2^ (%)	*χ* ^2^	*P* value	
All-cause mortality	3,887,585	28								
Age (years)										0.97
< 60	2,517,958	17	1.10	1.01	1.21	0.03	64.60	45.24	< 0.001	
≥ 60	268,044	7	1.10	1.04	1.18	< 0.001	0.00	3.00	0.81	
Others	1,101,583	4	1.19	0.91	1.57	0.21	84.30	19.05	< 0.001	
Location										0.63
Asia	1944.650	8	1.12	1.03	1.21	0.05	56.00	15.89	0.03	
Western Europe	347,906	8	1.18	0.93	1.50	0.18	76.40	29.69	< 0.001	
North America	1,572,948	8	1.10	1.08	1.12	< 0.001	0.00	4.33	0.74	
Others	22,081	4	0.96	0.73	1.26	0.77	32.80	4.47	0.22	
Follow-up years										0.64
< 10	908,252	9	1.12	1.02	1.22	0.02	38.00	12.91	0.12	
≥ 10	2,979,333	19	1.13	1.06	1.21	< 0.001	66.2	53.32	< 0.001	
Publication years										0.55
≤ 2000	95,532	9	1.1	0.9	1.4	0.5	64.50	22.51	< 0.001	
2001–2009	1,381,865	5	1.3	1.0	1.6	< 0.001	81.50	21.60	< 0.001	
≥ 2010	2,410,188	14	1.1	1.0	1.2	< 0.001	39.8	21.59	0.06	
Method of diabetes ascertainment										0.24
KDM	2,486,016	18	1.17	1.07	1.26	< 0.001	74.2	65.97	< 0.001	
NDM	590,506	6	1.05	0.90	1.21	0.20	32.10	7.36	0.6	
KDM, NDM	1,363,765	9	1.05	0.97	1.15	0.3	16.40	9.56	< 0.001	
Treated diabetes	NA									
Cancer mortality	4,482,501	14								
Age (years)										0.92
< 60	3,361,850	12	1.01	0.95	1.07	0.75	0.00	10.69	0.47	
≥ 60	66,820	1	1.02	0.88	1.18	0.81	NA	0.00	NA	
Others	52,655	1	1.04	0.99	1.09	0.17	NA	0.72	NA	
Location										0.56
Asia	2,795,136	8	1.01	0.96	1.08	0.65	0.00	5.33	0.62	
Western Europe	276,141	3	0.94	0.58	1.51	0.80	53.50	4.30	0.12	
North America	1,411,224	3	1.04	0.99	1.09	0.15	0.00	0.03	0.98	
Others	NA									
Follow-up years										0.47
< 10	881,061	3	1.08	0.94	1.23	0.29	0.00	1.45	0.49	
≥ 10	3,601,440	11	1.02	0.98	1.06	0.32	0.00	9.18	0.52	
Publication years										
≤ 2000	5131	1	1.11	0.31	3.94	0.87	NA	0.00	NA	0.73
2001–2009	1,327,437	2	1.05	0.91	1.20	0.50	5.10	1.05	0.31	
≥ 2010	3,149,933	11	1.02	0.98	1.06	0.30	0.00	9.99	0.44	
Method of diabetes ascertainment										0.72
KDM	2,094,903	9	1.03	0.90	1.19	0.65	77.90	36.21	< 0.001	
NDM	557,524	2	1.07	0.96	1.18	0.22	0.00	0.04	0.84	
KDM, NDM	2,369,318	4	1.03	0.99	1.08	0.16	0.00	1.16	0.764	
Treated diabetes	18,280	1	0.99	0.56	1.74	0.96	NA	0	NA	
CVD mortality	2,067,486	23								
Age (years)										0.91
< 60	867,999	18	1.26	1.01	1.56	0.04	72.20	61.20	< 0.001	
≥ 60	106,601	3	1.12	0.98	1.29	0.10	5.70	2.12	0.35	
Others	1,092,886	2	1.53	0.77	3.04	0.23	96.60	29.05	< 0.001	
Location										0.64
Asia	159,835	6	1.08	0.96	1.22	0.20	0.00	4.95	0.42	
Western Europe	460,756	8	1.49	1.17	1.90	< 0.001	58.70	16.96	0.02	
North America	1,415,878	6	1.33	1.03	1.72	0.03	88.20	42.22	< 0.001	
Others	31,017	3	1.12	0.75	1.67	0.57	0.00	0.53	0.77	
Follow-up years										0.38
< 10	433,100	6	1.08	0.96	1.22	0.19	0.00	4.13	0.53	
≥ 10	1,634,386	17	1.35	1.13	1.62	< 0.001	83.00	93.96	< 0.001	
Publication years										0.13
≤ 2000	54,288	4	1.36	0.75	2.47	0.31	79.10	14.33	< 0.001	
2001–2009	142,444	6	1.63	1.04	2.57	0.03	83.50	30.31	< 0.001	
≥ 2010	1,870,754	13	1.09	1.06	1.12	< 0.001	0.00	4.62	0.97	
Method of diabetes ascertainment										0.53
KDM	1,876,261	11	1.20	1.02	1.42	0.03	85.10	66.94	< 0.001	
NDM	42,944	3	1.40	0.84	2.35	0.20	74.50	7.85	0.02	
KDM, NDM	152,371	11	1.31	0.95	1.82	0.10	73.10	37.23	< 0.001	
Treated diabetes	NA									
CHD mortality	2,050,929	23								
Age (years)										0.88
< 60	864,790	15	1.52	1.22	1.90	< 0.001	39.20	23.02	0.06	
≥ 60	89,838	4	1.68	1.22	2.30	< 0.001	0.00	2.69	0.44	
Others	1,096,301	4	1.65	0.90	3.04	0.11	89.70	29.18	< 0.001	
Location										0.88
Asia	692,384	5	1.53	0.99	2.38	0.06	61.10	10.29	0.04	
Western Europe	242,624	8	1.86	1.42	2.45	< 0.001	41.60	11.98	0.10	
North America	1,113,375	9	1.17	1.13	1.22	< 0.001	0.00	7.26	0.51	
Others	2546	1	3.11	0.79	12.23	0.11	NA	0.00	NA	
Follow-up years										0.17
< 10	606,561	6	1.23	0.85	1.79	0.23	27.20	6.87	0.27	
≥ 10	1,371,125	14	1.75	1.33	2.31	0.00	78.50	60.43	< 0.001	
Others	73,243	3	1.38	0.95	2.02	0.10	0.00	0.86	0.65	
Publication years										0.20
≤ 2000	111,122	10	1.66	1.21	2.27	0.00	41.90	15.49	0.08	
2001–2009	118,915	6	1.84	1.25	2.71	0.00	32.80	7.44	0.19	
≥ 2010	1,820,892	7	1.30	1.12	1.52	0.00	41.00	10.17	0.12	
Method of diabetes ascertainment										0.85
KDM	1,457,769	14	1.52	1.20	1.92	0.00	78.10	59.23	< 0.001	
NDM	119,825	4	1.90	0.98	3.70	0.06	70.70	10.26	0.02	
KDM, NDM	543,435	7	1.34	1.14	1.57	0.00	0.00	5.42	0.49	
Treated diabetes	NA									
Stroke mortality	2,292,387	15								
Age (years)										0.71
< 60	1,078,421	10	1.12	0.98	1.28	0.11	0.00	7.36	0.60	
≥ 60	105,674	2	1.06	0.85	1.33	0.61	0.00	0.03	0.86	
Others	1,108,292	3	1.07	0.99	1.15	0.08	0.00	1.67	0.43	
Location										0.42
Asia	764,335	7	1.11	0.97	1.26	0.12	0.00	5.04	0.54	
Western Europe	132,562	5	1.36	0.97	1.90	0.07	0.00	1.60	0.81	
North America	1,392,944	2	1.06	0.98	1.13	0.13	0.00	0.08	0.78	
Others	2546	1	0.46	0.03	7.76	0.59	NA	0.00	NA	
Follow-up years										0.58
< 10	903,575	4	1.12	0.96	1.30	0.14	0.00	2.62	0.45	
≥ 10	1,388,812	11	1.07	1.00	1.14	0.06	0.00	6.48	0.77	
Others	NA									
Publication years										0.25
≤ 2000	67,444	5	1.39	0.90	2.14	0.14	0.00	2.67	0.62	
2001–2009	101,874	3	1.09	0.63	1.91	0.75	23.90	2.63	0.27	
≥ 2010	2,123,069	7	1.07	1.00	1.14	0.04	0.00	2.66	0.85	
Method of diabetes ascertainment										0.27
KDM	1,720,989	10	1.06	1.00	1.13	0.07	0.00	8.36	0.50	
NDM	61,368	2	1.37	0.70	2.66	0.36	24.80	1.33	0.25	
KDM, NDM	532,544	4	1.18	0.98	1.42	0.09	0.40	3.01	0.39	
Treated diabetes	NA									

*Abbreviations*: *N* number of studies, *NA* not available, *CVD* cardiovascular disease, *CHD* coronary heart disease

## Discussion

This systematic review and meta-analysis of 49 studies with 86 prospective cohorts found that diabetes conferred a greater risk for almost all outcomes of interest. Diabetes appears to be a stronger risk factor for CHD, CVD, and all-cause mortality in women than in men. Of note, compared to men with diabetes, women with the same condition had 57% excess risk for CHD. Although diabetes was associated with a higher risk of cancer mortality, infectious disease, and respiratory disease mortality, we did not observe a sex difference between diabetes and mortality. Interestingly, however, these results were only upheld in studies that used self-reporting measures to identify diabetes cases.

Diabetologists and epidemiologists have long been aware that diabetes has pronounced cardiovascular consequences for women, irrespective of diabetes type [10, 28, 64]. Indeed, CVD is the leading cause of morbidity and mortality for individuals with diabetes, which accounts for > 50% of all deaths [65]. We found that for women, diabetes confers a 54% excess risk of CHD death. While such sex-specific differences are of increasing interest in cardiology and medical fields, the underpinning mechanisms driving this association are not entirely clear. The pathogenesis seems to be multifactorial with contributions from sex differences in genetic and biological factors, gender disparities from cultural and environmental factors, and the well-documented differences in the diagnosis, management, and treatment of DM and CVD of women and men [66–68].

The putative biological mechanisms have centered on the effects of estrogen which can deplete during menopause to elevate women’s CHD risk [69]. Testosterone may be involved in different mechanisms attributed to sex differential in CHD risk [70–72]. In men, higher total testosterone levels are associated with reduced risk of future CHD and ischemic stroke. Testosterone has anabolic effects, promoting muscle mass and strength [73]. The recent prospective cohort study of half a million UK Biobank participants showed that higher grip strength was associated with a lower risk of incidence of and mortality from CVD [74]. Compared with men, women with lower testosterone levels have low mass and strength of muscle, which also partially explain greater risk for CHD death conferred by diabetes in women compared with men.

Women with diabetes are more likely to have poor risk factor profiles and suffer greater disease risk owing to the effects of individual risk factors. A recent meta-analysis showed that smoking conferred 25% excess risk for CHD in women than in men [7]. In addition, women with diabetes remain less likely to achieve high-density lipoprotein cholesterol targets and have a higher prevalence of obesity than men [75–77]. Whether existing sex differences in diabetic heart disease are magnified by sex differences in traditional and modifiable cardiac risk factors requires consideration. Recently, a meta-analysis of individual data from 68 prospective studies showed that body mass index, blood pressure, and total cholesterol each had continuous log-linear associations with CHD or stroke mortality that were similar in strength among those with and those without diabetes, irrespective of sex [78]. Our other study found that compared with men with metabolic syndrome, women with metabolic syndrome had a significant 16% higher risk of CHD incidence (RRR 1.16, 95% CI 1.01 to 1.34; *P* = 0.04), and the significant sex difference disappeared in non-diabetes population (RRR 0.92, 95% CI 0.73 to 1.17; *P* = 0.50). This partly supported the hypothesis that the stronger detrimental effects of diabetes for women than for men in CVD could not be explained by the different levels of established major CVD risk factors and their clusters. Differences in the clinical manifestation of diabetes warrants further consideration. Prediabetes is associated with an increased risk of cardiovascular disease [79], and the sex differences in the non-physiological effects can be partly accounted for the diabetes-related excess risk of CVD in women. In the prediabetic state, impaired glucose tolerance may be more serious in women than in men [80, 81].

Biases embedded within health service need to be considered. There is evidence that women, compared to their male counterparts, are less likely to have their risk factors assessed by physicians when they present in primary care. Compared to older women at high risk of CVD, younger women at high risk were less likely to receive preventative treatment [82]. Indeed, women with diabetes or CVD are diagnosed later and have a lower frequency of statin therapy, aspirin use, and ACE inhibitor and β-blocker use than men [83]. Some studies observed lower medication adherence in women than in men [84, 85]. Where medication is adhered to, women do not always benefit to the same extent as men given the well-documented issues with under-representation of women in clinical trials [66]. What is more, younger women’s symptoms often present differently to those of men of the same age. There may be less myocardial ischemic preconditioning in women, and subsequently greater susceptibility to ischemia. Therefore, sex and gender disparities in treatment may exacerbate the sex differences in CVD owing to diabetes [86, 87].

Some studies show that the proportion of undiagnosed diabetes to total diabetes in men is higher than that in women [88, 89]. In studies that used self-reported measures to identify diabetes, there was a greater proportion of undiagnosed diabetes in men. It is possible that this concealed the true excess risk of mortality conferred by diabetes in men and subsequent sex-specific relative risk estimates that were calculated for women and men.

Our finding that diabetes elevates the risk of all-cancer mortality is in general agreement with previous reviews [90]. However, most have looked at site-specific cancers; sex-specific associations from which results have been inconsistent. One meta-analysis indicated that diabetes conferred a stronger positive relationship with kidney cancer mortality and gastric cancer risk in women than in men [91, 92]. Others have found that diabetes increased the risk of esophageal cancer and leukemia in men, but not in women [93, 94]. Prospective studies showed that HRs for non-cancer, non-vascular deaths among participants with diabetes, as compared to those without diabetes, were also significantly higher among women (women: HR 2.20, 95% CI 1.91 to 2.52; men: HR 1.58, 95% CI 1.41 to 1.76; *P*_interaction_ < 0.001). The absence of sex disparities for infectious disease and respiratory disease mortality did not contribute to the sex difference for non-cancer, non-vascular deaths [95]. Therefore, future research is needed to distinguish whether and to what extent the excess risk of cause-specific mortality from non-cancer, non-vascular deaths conferred by diabetes differs between the sexes, such as kidney disease mortality.

### Strengths and limitations

The present meta-analysis has several main strengths. Firstly, the large number of participants ensured greater statistical power to detect sex differences than some previous individual studies. Secondly, using within-study comparisons to estimate sex-specific relationships between diabetes and cause-specific outcomes can minimize the role of extraneous, between-study factors. Thirdly, the study comprehensively evaluated the sex-specific associations for a range of important health outcomes: all-cause, all-cancer, CVD, and other cause-specific mortality. This has the potential to be more informative in aiding our understanding of the sex-specific burden of disease from diabetes. Fourthly, the detailed subgroup, sensitivity, and influence analyses ensure the robustness of the study findings.

There are also some specific limitations of this review that merit consideration. Firstly, there was some heterogeneity across studies for outcomes such as all-cause, CVD, and CHD mortality, but subgroup analyses and meta-regression analyses on study characteristics including age, geographical location, duration of follow-up, publish year, and method of diabetes ascertainment did not provide any evidence of a substantial effect of these differences on the results. Secondly, the present meta-analysis is based on prospective cohort studies, and the observational design is open to biases due to the residual confounding from incompletely measured factors and cannot elucidate causal relationship. Thirdly, the present meta-analysis did not include non-fatal events, which limited the ability to assess the presence of sex differences in risk for the incidence. Fourthly, differences in definition of diabetes, diabetes duration, duration of follow-up, and populations might have contributed to the sex differences in the association of diabetes with risk of death and CVD; although subgroup, meta-regression, and sensitivity analyses were conducted to explore the potential between-study heterogeneity, lack of individual participant data limited more in-depth sensitivity analyses than were reported here. Fifthly, our analysis cannot ascertain the underlying cause of the sex differences in the relationship between diabetes and the risk of CVD mortality. Finally, the potential publication bias was also a concern. Although we did not observe any apparent publication bias in our statistical tests, it was still difficult to completely rule this out.

## Conclusions

Our study demonstrated that women with diabetes have a greater risk of all-cause mortality, particularly from CHD, compared with men with the same condition. An increased understanding and appreciation of sex differences in the relationship between diabetes and risk of all-cause and CHD mortality is required given the substantial global and regional burden of NCDs. Women with diabetes should be treated and managed throughout their life course with the view to reduce the burden of other diseases related to diabetes. In the future, in-depth sex-specific analyses from randomized trials and other studies using approaches like Mendelian randomization are needed to clarify the biological, behavioral, or social mechanisms involved.

## Additional file

Additional file 1: contains additional information and analysis. **Table S1.** Study protocol: PRISMA 2009 Checklist. **Table S2.** Quality of included studies assessed with Newcastle-Ottawa Scale. **Figure S1.** Pooled RRs for the risk of cancer mortality. **Figure S2.** Pooled women-to-men RRRs for the risk of cancer mortality. **Figure S3.** Pooled RRs for the risk of CVD mortality. **Figure S4.** Pooled women-to-men RRRs for the risk of CVD mortality. **Figure S5.** Pooled RRs for the risk of CHD mortality. **Figure S6.** Pooled women-to-men RRRs for the risk of CHD mortality. **Figure S7.** Pooled women-to-men RRRs for the risk of stroke mortality. **Figure S8.** Pooled RRs for the risk of respiratory mortality. **Figure S9.** Pooled RRs for the risk of infectious mortality. (DOCX 1577 kb)

## Data Availability

All data and materials analyzed in this manuscript are authentic and derived from published studies except data from NHIS. For NHIS, the data we used are publicly available. The datasets used and/or analyzed during the current study are available from the corresponding author on reasonable request.

## Abbreviations

BMI: Body mass index
CHD: Coronary heart disease
CI: Confidence intervals
CVD: Cardiovascular disease
GBD: Global Burden of Disease Study
HR: Hazard ratio
KDM: Known diabetes
*N*: Number of studies
NA: Not available
NCDs: Non-communicable diseases
NDM: Newly diagnosed diabetes
NOS: Newcastle-Ottawa quality assessment scale
OR: Odds ratio
RR: Relative risk
RRR: Ratio of RR
