# Supplementary material for: Sex differences in the association between diabetes and risk of cardiovascular disease, cancer, and all-cause and cause-specific mortality: a systematic review and meta-analysis of 5,162,654 participants
Source: BMC Med. 2019 Jul 12;17:136. doi: 10.1186/s12916-019-1355-0 (PMC6625042; doi:10.1186/s12916-019-1355-0)
Supplement: Supplementary file 1 — contains additional information and analysis. Table S1. Study protocol: PRISMA 2009 Checklist. Table S2. Quality of included studies assessed with Newcastle-Ottawa Scale. Figure S1. Pooled RRs for the risk of cancer mortality. Figure S2. Pooled women-to-men RRRs for the risk of cancer mortality. Figure S3. Pooled RRs for the risk of CVD mortality. Figure S4. Pooled women-to-men RRRs for the risk of CVD mortality. Figure S5. Pooled RRs for the risk of CHD mortality. Figure S6. Pooled women-to-men RRRs for the risk of CHD mortality. Figure S7. Pooled women-to-men RRRs for the risk of stroke mortality. Figure S8. Pooled RRs for the risk of respiratory mortality. Figure S9. Pooled RRs for the risk of infectious mortality. (DOCX 1577 kb) [file 12916_2019_1355_MOESM1_ESM.docx]

**Sex differences in the association between diabetes and risk of cardiovascular disease, cancer, and all-cause and cause-specific mortality: a systematic review and meta-analysis of 5,162,654 participants**

Yafeng Wang^1^, Adrienne O’Neil^2^, Yurui Jiao^3^, Lijun Wang^4^, Jingxin Huang^5^, Yutao Lan^5^, Yikun Zhu^2*^, Chuanhua Yu^6*^

**Additional file 1:**

The following materials are included in the Online Supplemental Material.

1. Search strategies in PubMed and EMBASE.

2. Table S1-S2.

3. Figure S1-S9.

**Search strategies in PubMed**

#1 Search (((((((((((("Blood Glucose"[Text Word]) OR "Glucose Intolerance"[Text Word]) OR "Prediabetic State"[Text Word]) OR Hyperglycemia[Text Word]) OR "impaired fasting glucose"[Text Word]) OR "impaired glucose tolerance"[Text Word]) OR "pre-diabetes"[Text Word]) OR prediabetes[Text Word]) OR "borderline diabetes"[Text Word]) OR "higher risk of diabetes "[Text Word]) OR hemoglobin A1c [Text Word]) OR HbA1c [Text Word] OR ("IGT"[Text Word]) OR ("IFG"[Text Word]) OR ("diabetes*"[Text Word]) OR ("diabetic*"[Text Word]))

#2 Search (((all-cause mortality[MeSH Terms]) OR total mortality[MeSH Terms])) OR (((((((cardiovascular disease[MeSH Terms]) OR coronary artery disease[MeSH Terms]) OR coronary heart disease[MeSH Terms]) OR ischemic heart disease[MeSH Terms]) OR cerebrovascular disorder[MeSH Terms]) OR stroke[MeSH Terms]) OR neoplasm[MeSH Terms]) OR cancer[MeSH Terms]) OR infection[MeSH Terms]) OR respiratory disease [MeSH Terms])

#3 Search mortality[MeSH Terms] OR death[MeSH Terms]

#4 Search men[MeSH Terms] OR male[MeSH Terms]

#5 Search women[MeSH Terms] OR female[MeSH Terms]

#6 Search epidemiologic studies[MeSH Terms] OR epidemiologic[MeSH Terms] OR cohort[MeSH Terms] OR prospective[MeSH Terms]

#7 #1 AND #2 AND #3 AND #4 AND #5 AND #6 Filters: Humans

**Search strategies in Embase**

#1 'glucose blood level' OR 'glucose intolerance' OR 'impaired glucose tolerance' OR 'hyperglycemia' OR 'diabetes mellitus'/exp OR 'diabetes mellitus'

#2 'mortality' OR 'cause of death' OR 'cardiovascular disease' OR 'malignant neoplasm'

#3 'female' AND 'male'

#4 #1 AND #2 AND #3

#5 #4 AND 'prospective study'/de

Additional file 1: Table S1: PRISMA 2009 Checklist

| **Section/topic** | **#** | **Checklist item** | **Reported on page #** |
| --- | --- | --- | --- |
| **TITLE** | | |  |
| Title | 1 | Identify the report as a systematic review, meta-analysis, or both. | 1 |
| **ABSTRACT** | | |  |
| Structured summary | 2 | Provide a structured summary including, as applicable: background; objectives; data sources; study eligibility criteria, participants, and interventions; study appraisal and synthesis methods; results; limitations; conclusions and implications of key findings; systematic review registration number. | 2 |
| **INTRODUCTION** | | |  |
| Rationale | 3 | Describe the rationale for the review in the context of what is already known. | 4 |
| Objectives | 4 | Provide an explicit statement of questions being addressed with reference to participants, interventions, comparisons, outcomes, and study design (PICOS). | 4 |
| **METHODS** | | |  |
| Protocol and registration | 5 | Indicate if a review protocol exists, if and where it can be accessed (e.g., Web address), and, if available, provide registration information including registration number. | 5 |
| Eligibility criteria | 6 | Specify study characteristics (e.g., PICOS, length of follow-up) and report characteristics (e.g., years considered, language, publication status) used as criteria for eligibility, giving rationale. | 5 |
| Information sources | 7 | Describe all information sources (e.g., databases with dates of coverage, contact with study authors to identify additional studies) in the search and date last searched. | 5 |
| Search | 8 | Present full electronic search strategy for at least one database, including any limits used, such that it could be repeated. | 5 |
| Study selection | 9 | State the process for selecting studies (i.e., screening, eligibility, included in systematic review, and, if applicable, included in the meta-analysis). | 5 |
| Data collection process | 10 | Describe method of data extraction from reports (e.g., piloted forms, independently, in duplicate) and any processes for obtaining and confirming data from investigators. | 5 |
| Data items | 11 | List and define all variables for which data were sought (e.g., PICOS, funding sources) and any assumptions and simplifications made. | 5 |
| Risk of bias in individual studies | 12 | Describe methods used for assessing risk of bias of individual studies (including specification of whether this was done at the study or outcome level), and how this information is to be used in any data synthesis. | 5,6 |
| Summary measures | 13 | State the principal summary measures (e.g., risk ratio, difference in means). | 6 |
| Synthesis of results | 14 | Describe the methods of handling data and combining results of studies, if done, including measures of consistency (e.g., I^2^) for each meta-analysis. | 6 |
| Risk of bias across studies | 15 | Specify any assessment of risk of bias that may affect the cumulative evidence (e.g., publication bias, selective reporting within studies). | 6 |
| Additional analyses | 16 | Describe methods of additional analyses (e.g., sensitivity or subgroup analyses, meta-regression), if done, indicating which were pre-specified. | 6 |
| **RESULTS** | | |  |
| Study selection | 17 | Give numbers of studies screened, assessed for eligibility, and included in the review, with reasons for exclusions at each stage, ideally with a flow diagram. | 7 |
| Study characteristics | 18 | For each study, present characteristics for which data were extracted (e.g., study size, PICOS, follow-up period) and provide the citations. | 7 |
| Risk of bias within studies | 19 | Present data on risk of bias of each study and, if available, any outcome level assessment (see item 12). | 7,8 |
| Results of individual studies | 20 | For all outcomes considered (benefits or harms), present, for each study: (a) simple summary data for each intervention group (b) effect estimates and confidence intervals, ideally with a forest plot. | 7,8 |
| Synthesis of results | 21 | Present results of each meta-analysis done, including confidence intervals and measures of consistency. | 7-9 |
| Risk of bias across studies | 22 | Present results of any assessment of risk of bias across studies (see Item 15). | 9 |
| Additional analysis | 23 | Give results of additional analyses, if done (e.g., sensitivity or subgroup analyses, meta-regression [see Item 16]). | 9 |
| **DISCUSSION** | | |  |
| Summary of evidence | 24 | Summarize the main findings including the strength of evidence for each main outcome; consider their relevance to key groups (e.g., healthcare providers, users, and policy makers). | 9-13 |
| Limitations | 25 | Discuss limitations at study and outcome level (e.g., risk of bias), and at review-level (e.g., incomplete retrieval of identified research, reporting bias). | 12,13 |
| Conclusions | 26 | Provide a general interpretation of the results in the context of other evidence, and implications for future research. | 13 |
| **FUNDING** | | |  |
| Funding | 27 | Describe sources of funding for the systematic review and other support (e.g., supply of data); role of funders for the systematic review. | 14 |

| Additional file 1: Table S2. Quality of included studies assessed with Newcastle-Ottawa Scal**e** | | | | | | | | | |
| --- | --- | --- | --- | --- | --- | --- | --- | --- | --- |
| **Author** | **Selection** | | | | **Comparability** | **Outcome** | | | **Overall quality** |
|  | **Representative of the exposed cohort** | **Selection of the non-exposed cohort** | **Exposure ascertainment** | **Demonstration that outcome of interest was not present at start of study** | **Comparability of cohorts** | **Outcome assessment** | **Adequate follow-up time (> 5 years)** | **Adequacy of follow up of cohorts** |  |
| Jousilahti et al, 1999 | 1 | 1 | 0 | 1 | 2 | 1 | 0 | 0 | 6 |
| Oba et al, 2008 | 1 | 1 | 0 | 1 | 1 | 1 | 1 | 0 | 6 |
| Hu G et al, 2005 | 1 | 1 | 0 | 1 | 2 | 0 | 1 | 1 | 7 |
| Madssen et al, 2012 | 1 | 1 | 1 | 1 | 2 | 1 | 0 | 0 | 7 |
| DECODE Study Group, 2001 | 1 | 1 | 1 | 1 | 2 | 1 | 1 | 0 | 8 |
| Keli et al, 1993 | 1 | 1 | 0 | 0 | 2 | 1 | 1 | 0 | 6 |
| Friberg et al, 2004 | 1 | 1 | 1 | 1 | 2 | 1 | 1 | 1 | 9 |
| VR et al, 1996 | 1 | 1 | 1 | 1 | 2 | 1 | 1 | 0 | 8 |
| Bozorgmanesh et al, 2011 | 1 | 1 | 1 | 0 | 2 | 1 | 1 | 0 | 7 |
| Kleinman et al, 1988 | 1 | 1 | 1 | 1 | 2 | 1 | 1 | 0 | 8 |
| Magliano et al, 2010 | 1 | 1 | 1 | 1 | 2 | 0 | 1 | 0 | 7 |
| Elizabeth et al, 1991 | 1 | 1 | 1 | 1 | 2 | 1 | 1 | 0 | 8 |
| Fraser et al, 1992 | 1 | 1 | 0 | 1 | 2 | 0 | 1 | 1 | 7 |
| Sievers et al, 1992 | 1 | 1 | 1 | 1 | 1 | 1 | 1 | 0 | 7 |
| Seeman et al, 1993 | 0 | 1 | 0 | 1 | 2 | 1 | 1 | 0 | 6 |
| Campbell et al, 2012 | 1 | 1 | 0 | 1 | 2 | 1 | 1 | 0 | 7 |
| Wang et al, 2012 | 1 | 1 | 1 | 0 | 2 | 0 | 1 | 0 | 6 |
| Natarajan et al, 2003 | 1 | 1 | 1 | 0 | 2 | 0 | 1 | 0 | 6 |
| Vilbergsson et al, 1998 | 1 | 1 | 1 | 1 | 2 | 1 | 1 | 0 | 8 |
| Qvist et al, 1996 | 1 | 1 | 0 | 0 | 2 | 1 | 1 | 1 | 7 |
| Tunstall-Pedoe et al, 1997 | 1 | 1 | 0 | 1 | 2 | 0 | 1 | 0 | 6 |
| Nilsson et al, 1998 | 1 | 1 | 0 | 1 | 1 | 1 | 1 | 1 | 7 |
| Imazu et al, 2002 | 1 | 1 | 1 | 1 | 2 | 1 | 1 | 1 | 9 |
| Hart et al, 1999 | 1 | 1 | 0 | 1 | 2 | 1 | 1 | 0 | 7 |
| Bragg et al, 2014 | 1 | 1 | 1 | 1 | 2 | 1 | 1 | 1 | 9 |
| Kato et al, 2015 | 1 | 1 | 0 | 1 | 2 | 1 | 1 | 1 | 8 |
| Johansen et al, 1987 | 0 | 1 | 0 | 1 | 2 | 1 | 1 | 0 | 6 |
| Suemoto et al, 2014 | 1 | 1 | 0 | 1 | 2 | 1 | 0 | 0 | 6 |
| Jee et al, 2005 | 0 | 1 | 0 | 1 | 2 | 1 | 1 | 1 | 7 |
| Fraser et al, 1997 | 1 | 1 | 0 | 1 | 2 | 1 | 1 | 1 | 8 |
| Moe et al, 2013 | 1 | 1 | 1 | 1 | 2 | 1 | 1 | 0 | 8 |
| Liu et al, 2011 | 1 | 1 | 0 | 1 | 2 | 0 | 1 | 1 | 7 |
| Vimalananda et al, 2014 | 1 | 1 | 0 | 1 | 2 | 0 | 1 | 0 | 6 |
| Eichner et al, 2010 | 1 | 1 | 0 | 1 | 2 | 0 | 1 | 0 | 6 |
| Bozorgmanesh et al, 2012 | 1 | 1 | 1 | 0 | 2 | 0 | 1 | 0 | 6 |
| Moe et al, 2013 | 1 | 1 | 0 | 1 | 2 | 1 | 1 | 0 | 7 |
| Kakehi et al, 2014 | 1 | 1 | 1 | 1 | 2 | 1 | 1 | 1 | 9 |
| Shen et al, 2014 | 0 | 1 | 1 | 1 | 2 | 1 | 1 | 1 | 8 |
| Hiltunen et al, 2005 | 1 | 1 | 0 | 1 | 2 | 1 | 1 | 0 | 7 |
| Gordon-Dseagu et al, 2014 | 1 | 1 | 0 | 1 | 2 | 1 | 1 | 1 | 8 |
| Yeh et al, 2012 | 1 | 1 | 1 | 1 | 2 | 1 | 1 | 0 | 8 |
| Chen et al, 2017 | 1 | 1 | 0 | 1 | 2 | 1 | 1 | 1 | 8 |
| Zhou et al, 2010 | 1 | 1 | 1 | 1 | 2 | 1 | 1 | 1 | 9 |
| Drake et al, 2017 | 1 | 1 | 0 | 1 | 2 | 1 | 1 | 1 | 8 |
| Preis et al, 2009 | 1 | 1 | 0 | 1 | 1 | 1 | 1 | 1 | 7 |
| NHIS, 2018 | 1 | 1 | 1 | 1 | 2 | 1 | 1 | 1 | 9 |
| Natarajan et al, 2005 | 1 | 1 | 0 | 1 | 2 | 1 | 1 | 0 | 7 |
| Hirakawa et al, 2017 | 1 | 1 | 1 | 1 | 2 | 1 | 1 | 1 | 9 |
| Alegre-Díaz et al, 2016 | 1 | 1 | 1 | 1 | 2 | 1 | 1 | 1 | 9 |

Additional file 1:
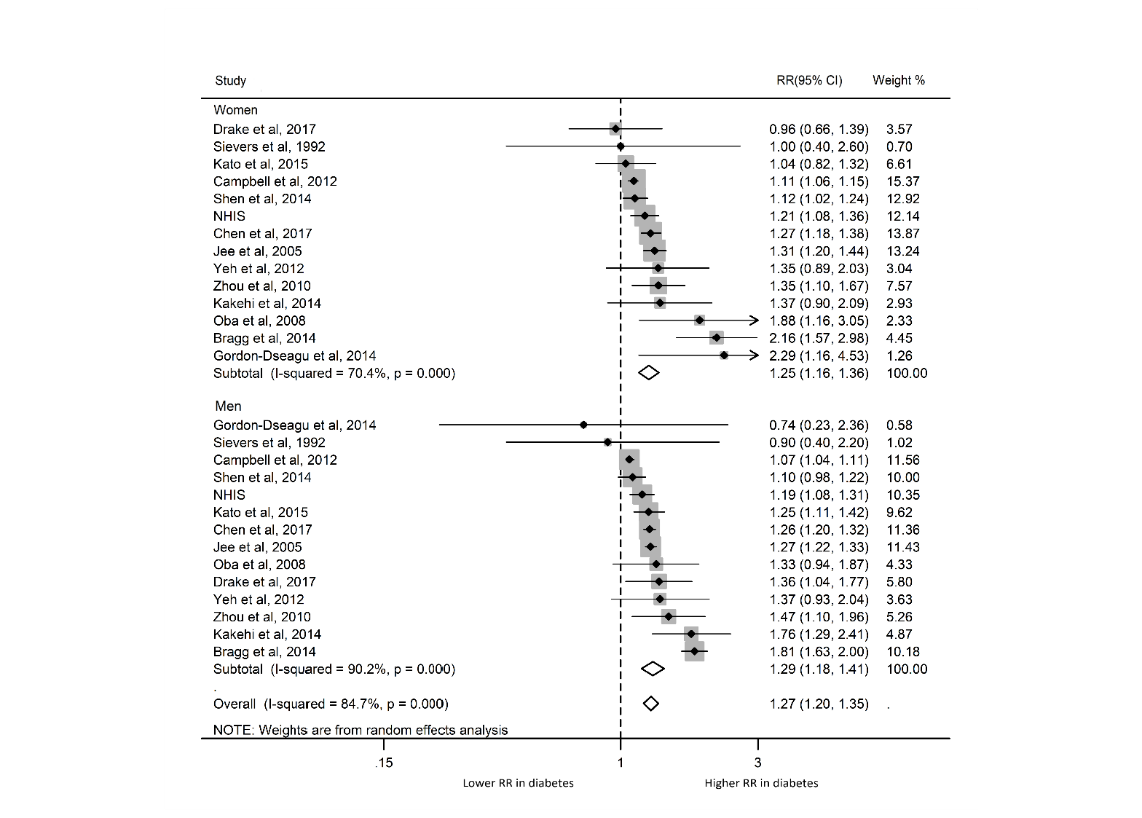
Figure S1. Pooled RRs for risk of cancer mortality; Abbreviations: RR: relative risk.

**
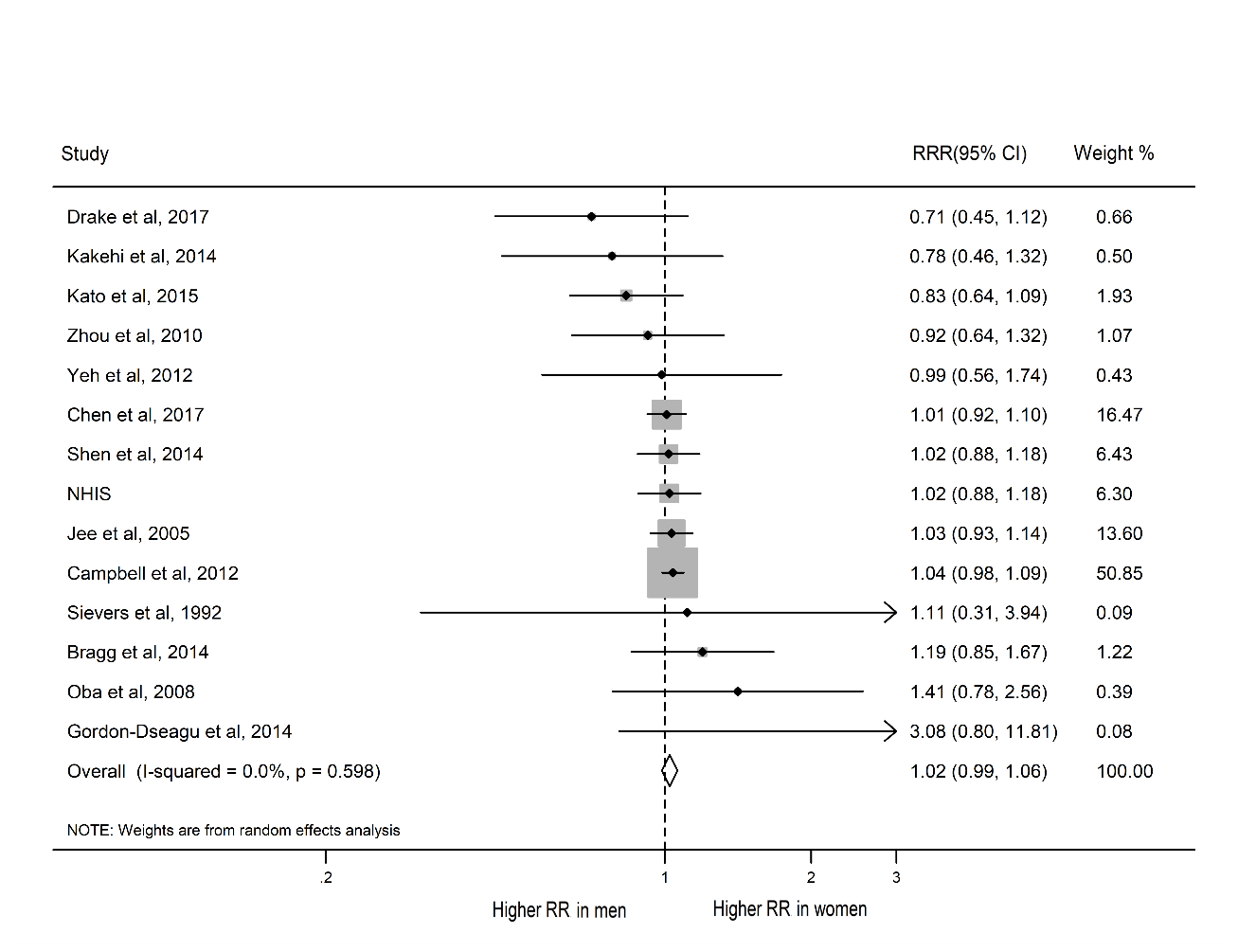
**

Additional file 1: Figure S2. Pooled women-to-men RRRs for risk of cancer mortality; Abbreviations: RR: relative risk; RRR: ratio of RR.

**
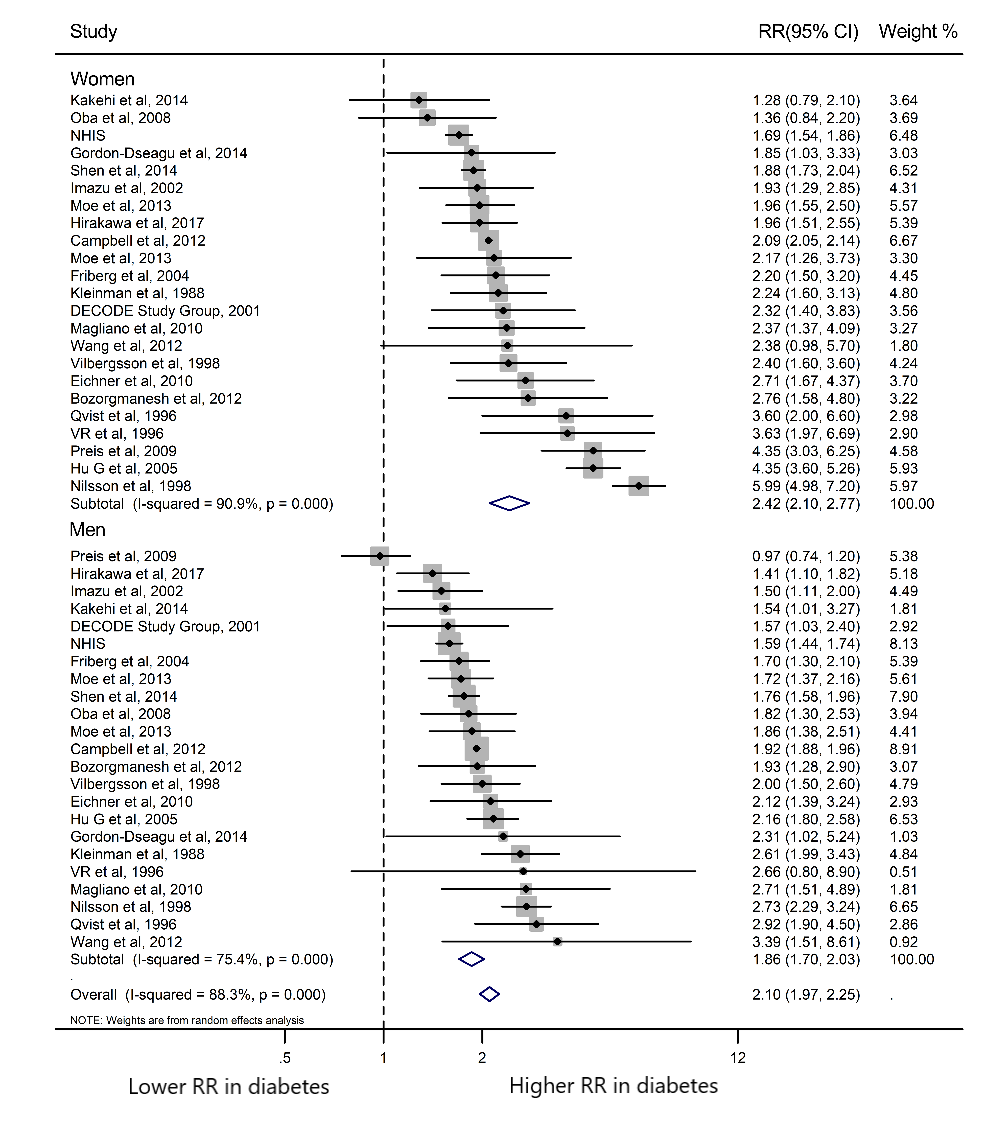
**

Additional file 1: Figure S3. Pooled RRs for risk of CVD mortality; Abbreviations: CVD: cardiovascular disease; RR: relative risk.


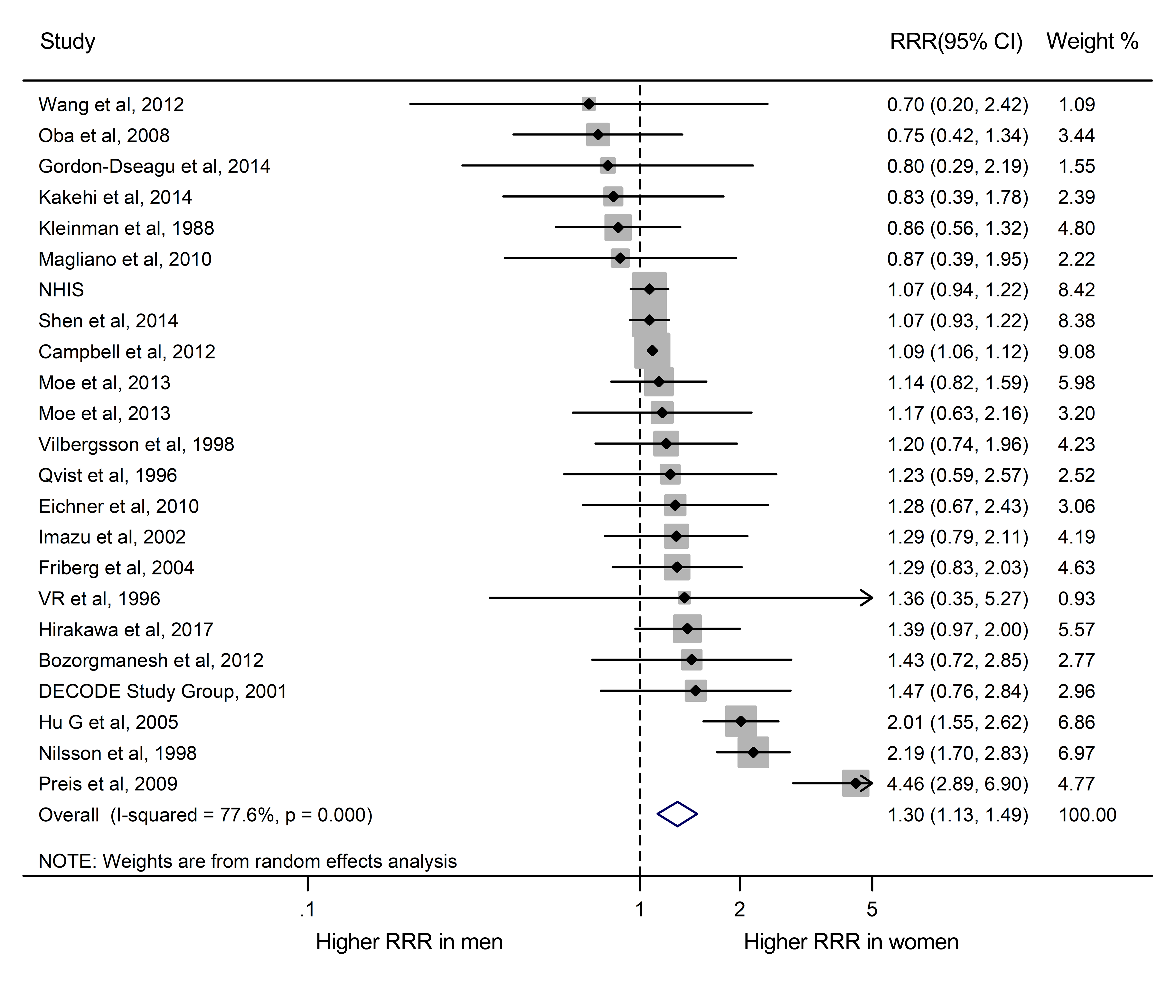


Additional file 1: Figure S4. Pooled women-to-men RRRs for risk of CVD mortality; Abbreviations: CVD: cardiovascular disease; RR: relative risk; RRR: ratio of RR.


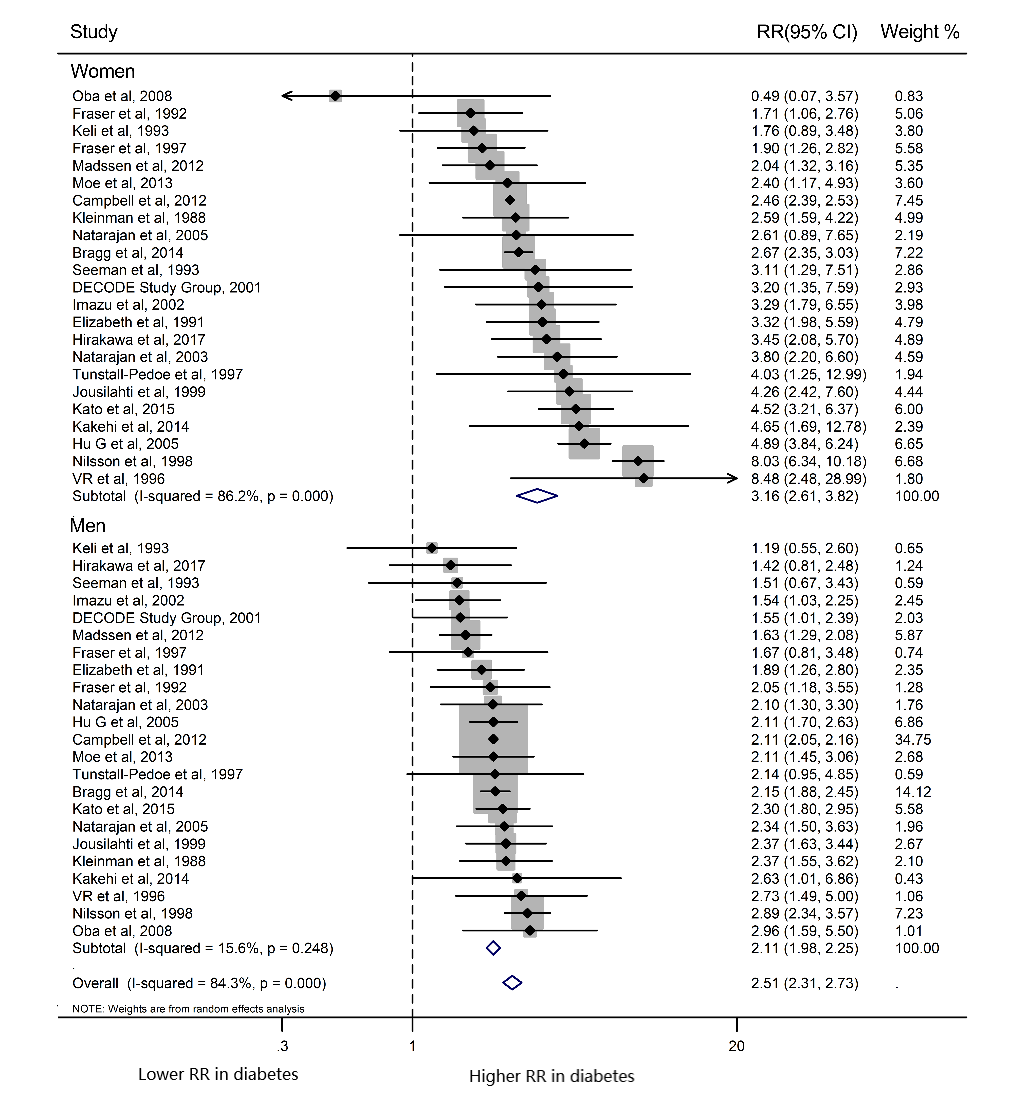
Additional file 1: Figure S5. Pooled RRs for risk of CHD mortality; Abbreviations: CHD: coronary heart disease; RR: relative risk.

**
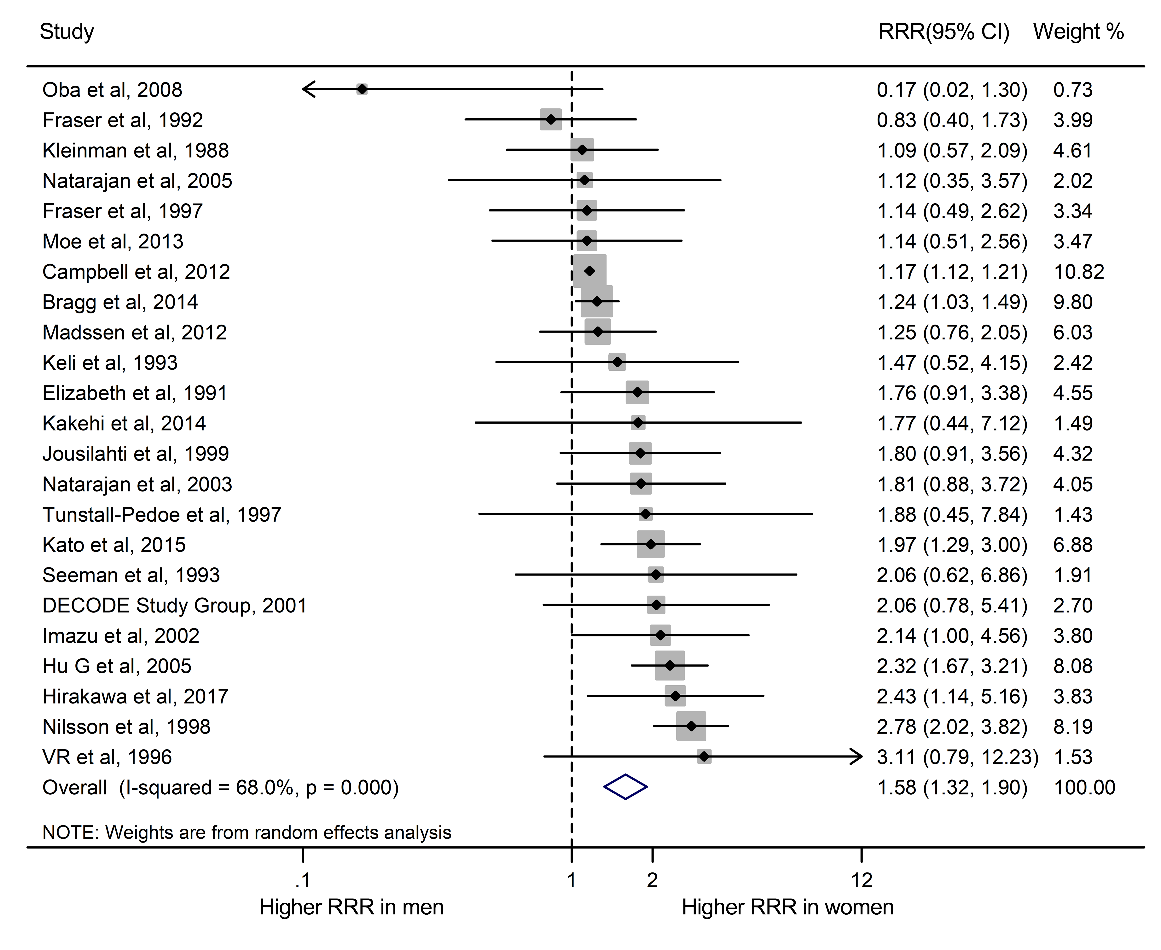
**

Additional file 1: Figure S6. Pooled women-to-men RRRs for risk of CHD mortality; Abbreviations: CHD: coronary heart disease; RR: relative risk; RRR: ratio of RR.

Additional file 1:
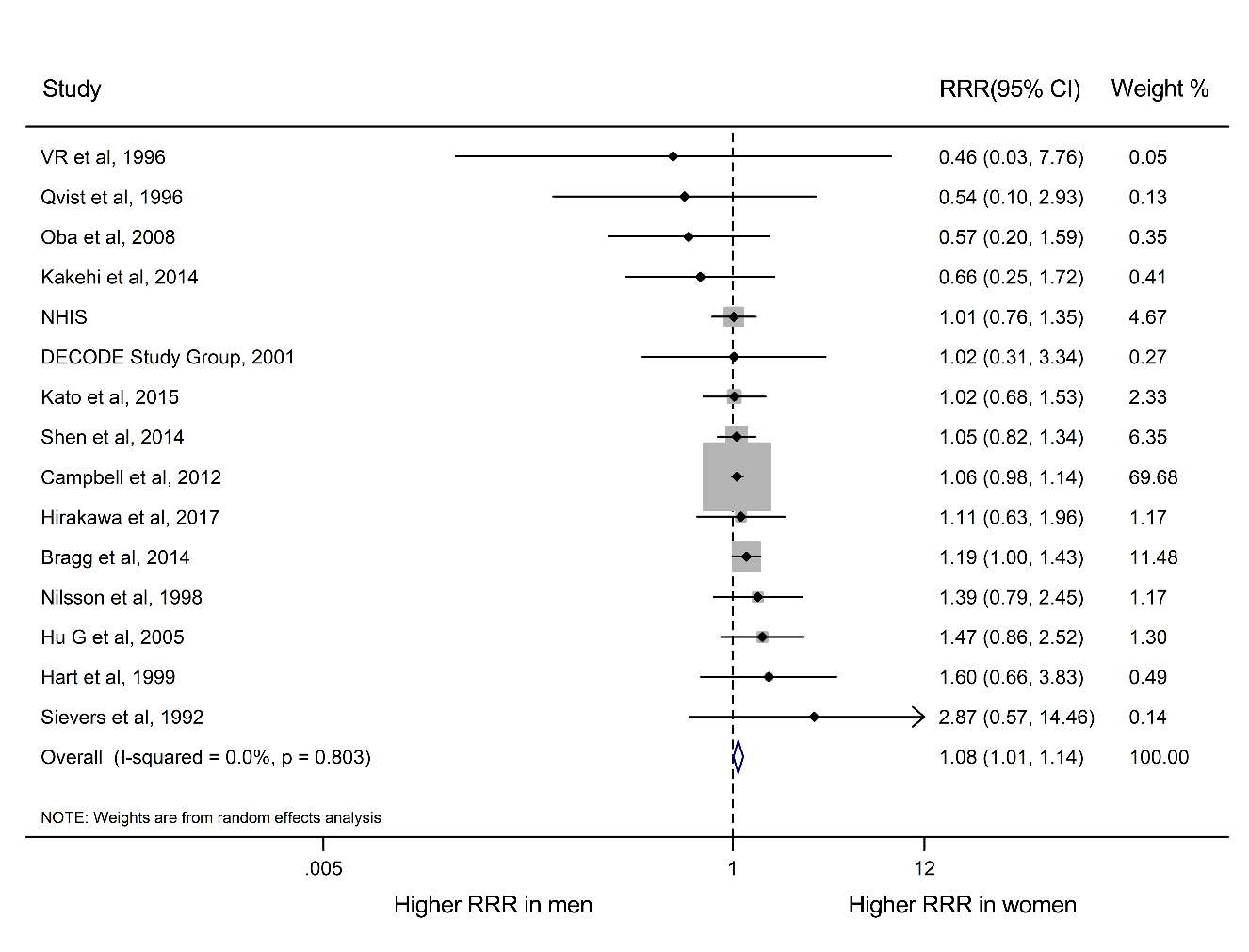
Figure S7. Pooled women-to-men RRRs for risk of stroke mortality; Abbreviations: RR: relative risk; RRR: ratio of RR.


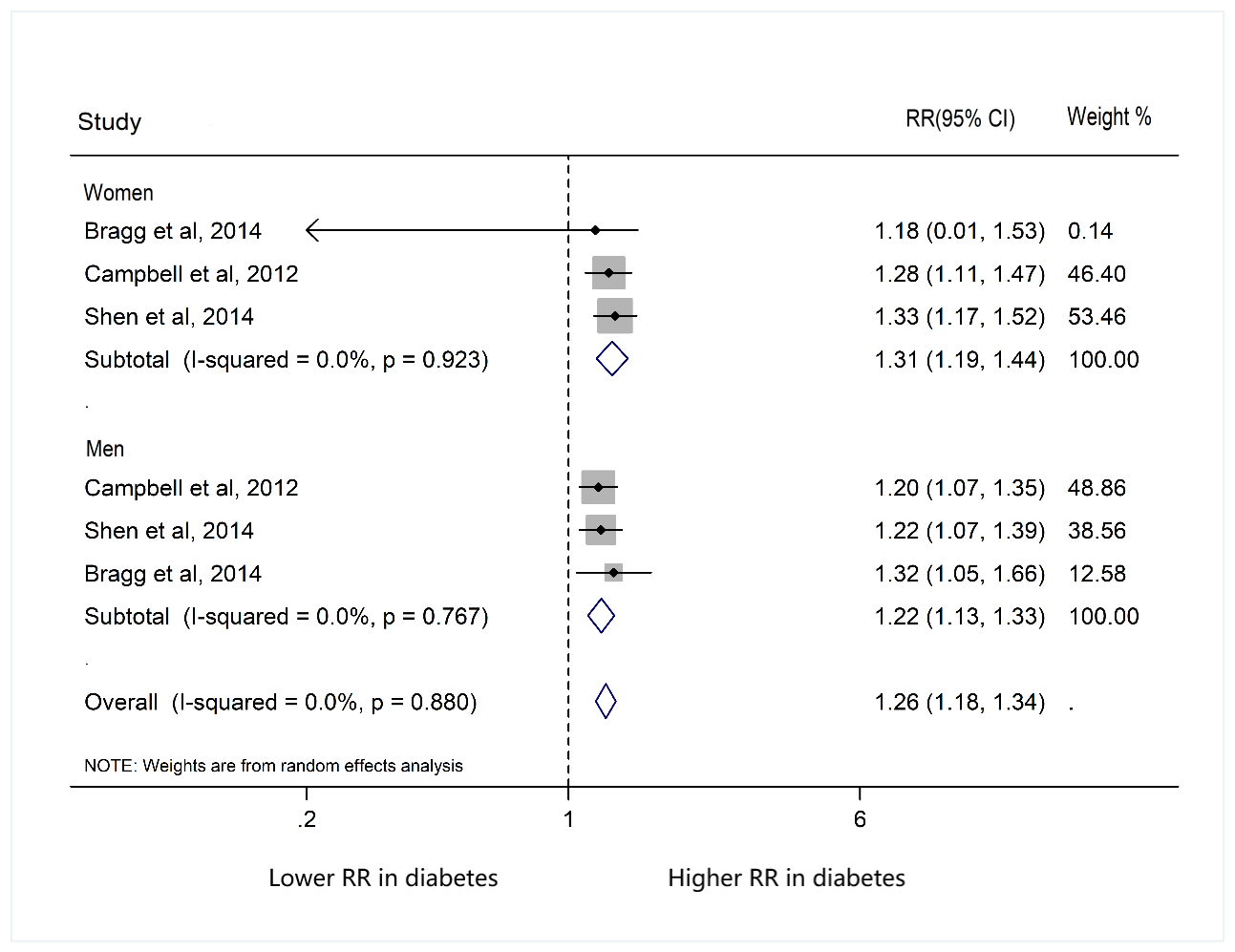
Additional file 1: Figure S8. Pooled RRs for risk of respiratory mortality; Abbreviations: RR: relative risk.

**
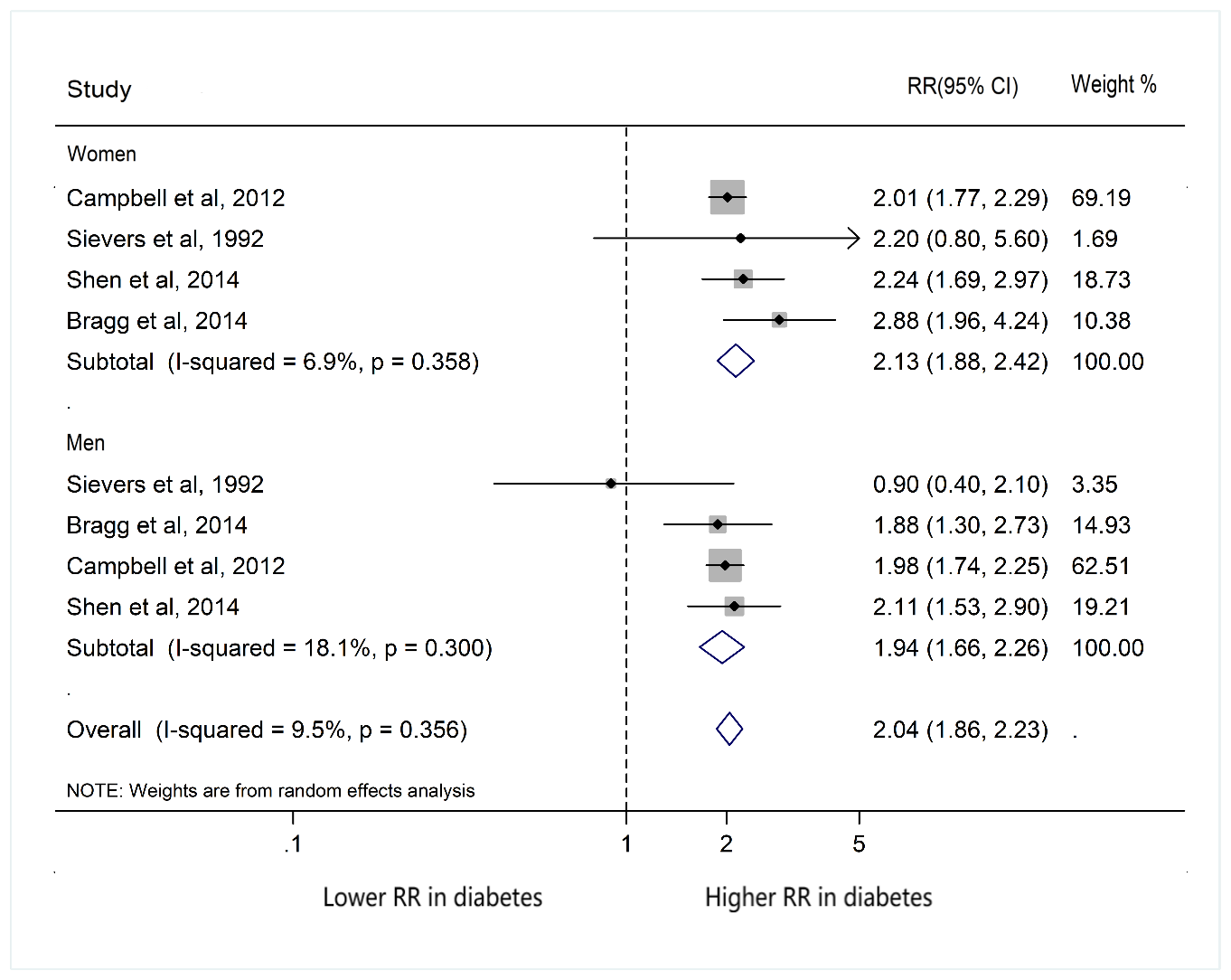
**

Additional file 1:
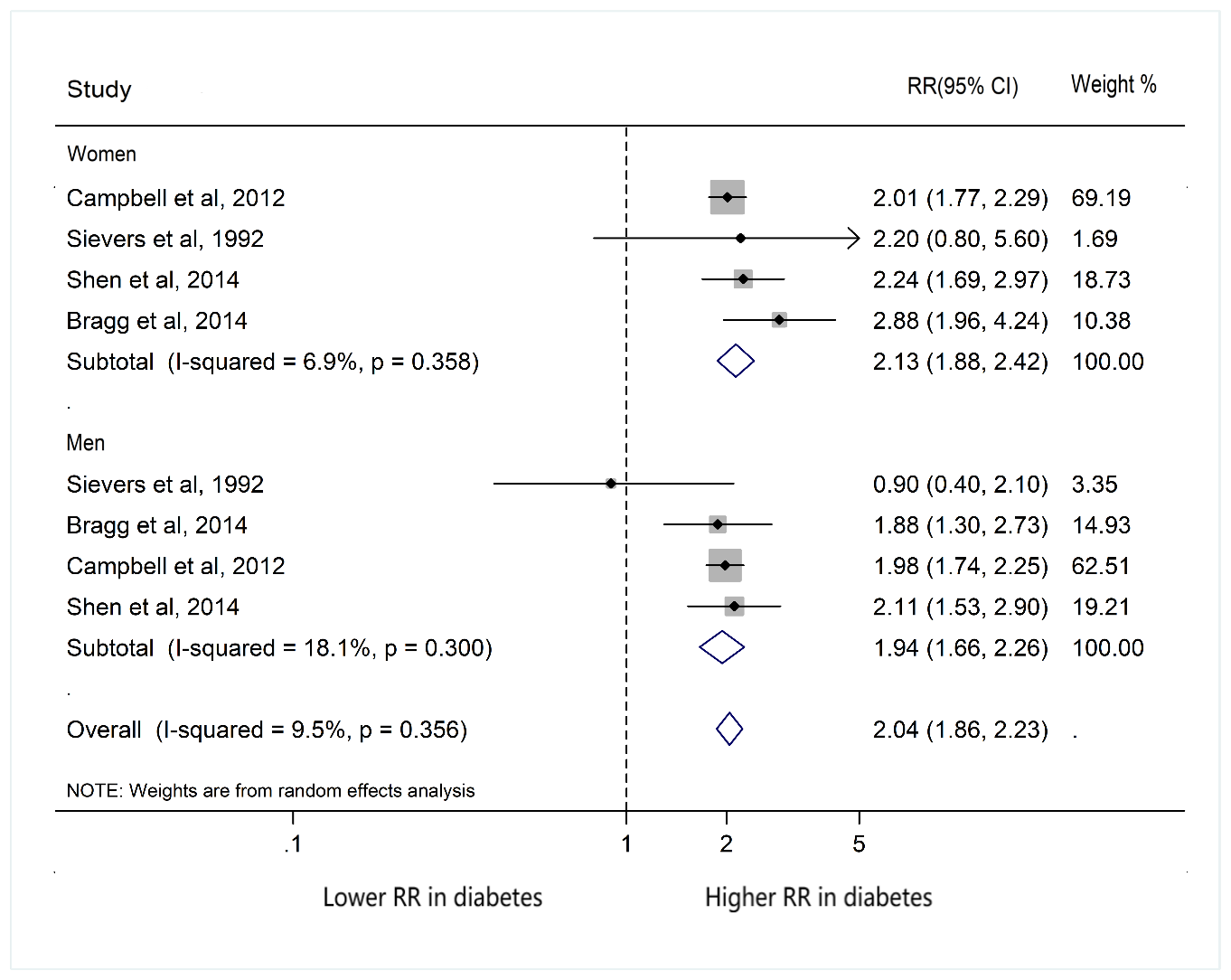
Figure S9. Pooled RRs for risk of infectious mortality; Abbreviations: RR: relative risk.
